# Serine/Threonine Kinase 33 as a Novel Target of Bufalin in Treatment of Triple‐Negative Breast Cancer

**DOI:** 10.1002/advs.202506253

**Published:** 2025-09-04

**Authors:** Shilong Jiang, Junyan Liu, Hui Li, Chan Zou, Xiaoya Wan, Rong Gong, Ting Jiang, Changxin Zhong, Zonglin Chen, Zewu Zhu, Dongsheng Cao, Yan Cheng

**Affiliations:** ^1^ Department of Pharmacy The Second Xiangya Hospital Central South University Changsha Hunan 410011 China; ^2^ Department of Pharmacy Xiangya Hospital Central South University Changsha Hunan 410008 China; ^3^ Xiangya School of Pharmaceutical Sciences Central South University Changsha Hunan 410013 China; ^4^ Department of Orthopaedics The Third Xiangya Hospital Central South University Changsha 410013 China; ^5^ Center for Clinical Pharmacology the Third Xiangya Hospital Central South University Changsha Hunan 410013 China; ^6^ Department of General Surgery The Second Xiangya Hospital Central South University Changsha Hunan 410011 China; ^7^ Hunan Provincial Engineering Research Centre of Translational Medicine and Innovative Drug Changsha Hunan 410011 China; ^8^ The Hunan Institute of Pharmacy Practice and Clinical Research Xiangya Hospital Central South University Changsha Hunan 410008 China; ^9^ Department of Urology Xiangya Hospital Central South University Changsha Hunan 410008 China; ^10^ FuRong Laboratory Changsha Hunan 410078 China; ^11^ NHC Key Laboratory of Cancer Proteomics & State Local Joint Engineering Laboratory for Anticancer Drugs Xiangya Hospital Central South University Changsha Hunan 410008 China

**Keywords:** Bufalin, STK33, triple‐negative breast cancer, SPR‐LC‐MS/MS, CCAR1

## Abstract

Identifying novel therapeutic targets and drugs is crucial for treating triple‐negative breast cancer (TNBC). Bufalin, a key active ingredient of the traditional Chinese medicine *HuaChansu*, has been employed in tumor therapy. Here, SPR‐LC‐MS/MS is employed to characterize the targets of Bufalin and found that serine/threonine kinase 33 (STK33) possesses a strong binding affinity to Bufalin. Combining molecular docking, SPR analysis, and Biotin‐pulldown analysis, it is demonstrated that STK33 can bind Bufalin. Notably, STK33 is highly expressed in TNBC and is associated with poor prognosis in TNBC patients. STK33 knockdown inhibits TNBC cell growth both in vitro and in vivo. Mechanistically, STK33 phosphorylates and stabilizes CCAR1, which promotes tumor growth and metastasis, thereby driving tumor progression. Further analyses confirmed that Methionine 245 of STK33 is required for STK33‐Bufalin interaction, and Bufalin treatment promotes the degradation of STK33 protein by destroying the STK33‐HSP90 complex. Through in vitro, in vivo, and in patient‐derived TNBC organoids, it is observed that Bufalin inhibited the TNBC cell proliferation by targeting STK33. This study not only establishes Bufalin as a putative STK33 degrader to suppress TNBC but also identifies STK33 as a pro‐cancer factor in TNBC, presenting a potential therapeutic target for TNBC.

## Graphical abstract

This study identified that STK33 may serve as a target of Bufalin, which induces the degradation of STK33 and inhibits the growth of TNBC. Furthermore, STK33 is highly expressed in TNBC and enhances TNBC cell proliferation by phosphorylating and stabilizing CCAR1. This study presents a promising therapeutic target and an anti‐tumor strategy for the management of TNBC.

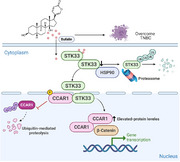

## 1 Introduction

Breast cancer is the most common and fatal cancer affecting women worldwide.^[^ ^1^ ^]^ Triple‐negative breast cancer (TNBC), which manifests as the lack of estrogen receptor (ER), progesterone receptor (PR) and human epidermal growth factorreceptor‐2 (HER2) (making it ER‐, PR‐, and HER2‐negative),^[^ ^1^, ^2^ ^]^ is a complex and recurrent cancer with a high risk of metastasis and poor prognosis.^[^ ^3^ ^]^ Unlike the various subtypes of breast cancer, TNBC presents with a higher mortality rate of 40%, especially when the disease is in the advanced stages within the first five years after diagnosis.^[^ ^4^, ^5^ ^]^ Although target‐based therapeutic options have been developed for the management of other cancers, there are few effective therapeutic options for TNBC. Therefore, novel and effective therapies are needed for TNBC.

Natural compounds serve as valuable resources of anticancer drugs, and identifying their specific binding proteins is a critical approach for discovering effective drug targets.^[^ ^6^, ^7^ ^]^ For instance, the identification of N‐Ethylmaleimide‐sensitive fusion (NSF) as a target for rhodojaponin VI has opened new avenues for developing analgesics for neuropathic pain.^[^ ^6^ ^]^ Similarly, identifying the proteins that specifically bind to anti‐tumor natural products in TNBC may provide effective targets for the treatment of TNBC. Bufalin, the major ingredient of the traditional Chinese medicine *HuaChansu*, has been applied for the treatment of various cancers.^[^ ^8^ ^]^ Accumulating evidence indicates that Bufalin exhibits anti‐tumor efficacy against a broad spectrum of cancers, including TNBC, and can reverse acquired drug resistance.^[^ ^9^, ^10^ ^]^ In addition, there is evidence that Bufalin can promote ferroptosis and modulate immune responses, resulting in suppression of cancer development.^[^ ^11^, ^12^ ^]^ Bufalin suppresses tumor cell proliferation via multiple signal pathways, such as PI3K‐Akt, Hippo‐YAP, MAPK, JNK, Wnt/β‐Catenin, TGF‐β/Smad, integrated vegetarian signaling pathway, and NF‐κB signaling pathway.^[^ ^13^, ^14^, ^15^, ^16^ ^]^ Despite these findings, the precise mechanisms by which Bufalin exerts its effects on cancer cells remain incompletely understood.

Serine/threonine kinase 33 (STK33) belongs to the calcium/calmodulin‐dependent kinases family. Data indicates that it is upregulated in various cancers such as hypopharyngeal squamous cell carcinoma,^[^ ^17^ ^]^ lung cancer,^[^ ^18^ ^]^ hepatocellular carcinoma,^[^ ^19^ ^]^ diffuse large B‐cell lymphoma,^[^ ^20^ ^]^ colorectal cancer,^[^ ^21^ ^]^ and pancreatic cancer.^[^ ^22^ ^]^ Numerous investigations have demonstrated that STK33 promotes tumor development and progression by regulating cell proliferation, differentiation, apoptosis, and DNA replication.^[^ ^19^, ^23^ ^]^ In addition, it was found to be overexpressed in cisplatin‐resistant diffuse large B‐cell lymphoma cells, and knockdown of STK33 significantly promoted the sensitivity of resistant cells to cisplatin.^[^ ^20^ ^]^ However, the functional role and molecular mechanisms of STK33 in breast cancer pathogenesis remain poorly understood. Given its established association with tumorigenesis, developing potent STK33 inhibitors represents a promising therapeutic strategy for targeted treatment of cancer.^[^ ^24^, ^25^, ^26^ ^]^

In this study, we reveal that STK33 is a putative target of Bufalin in TNBC. Moreover, the results indicate strong binding ability of Bufalin with STK33 at the Methionine 245 site and disruption of the STK33‐HSP90 complex by Bufalin, resulting in the ubiquitination and degradation of STK33 protein. STK33 is upregulated in TNBC, and knocking down STK33 inhibits TNBC growth in vitro and in vivo. Mechanistically, STK33 phosphorylates and increases the CCAR1 stability, thereby stimulating tumor progression. In summary, this study uncovers the underlying molecular mechanism by which Bufalin acts against TNBC and provides a potential novel target for the treatment of TNBC.

## 2 Results

### 2.1 Identification of STK33 as a Binding Protein of Bufalin

**Figure 1:**
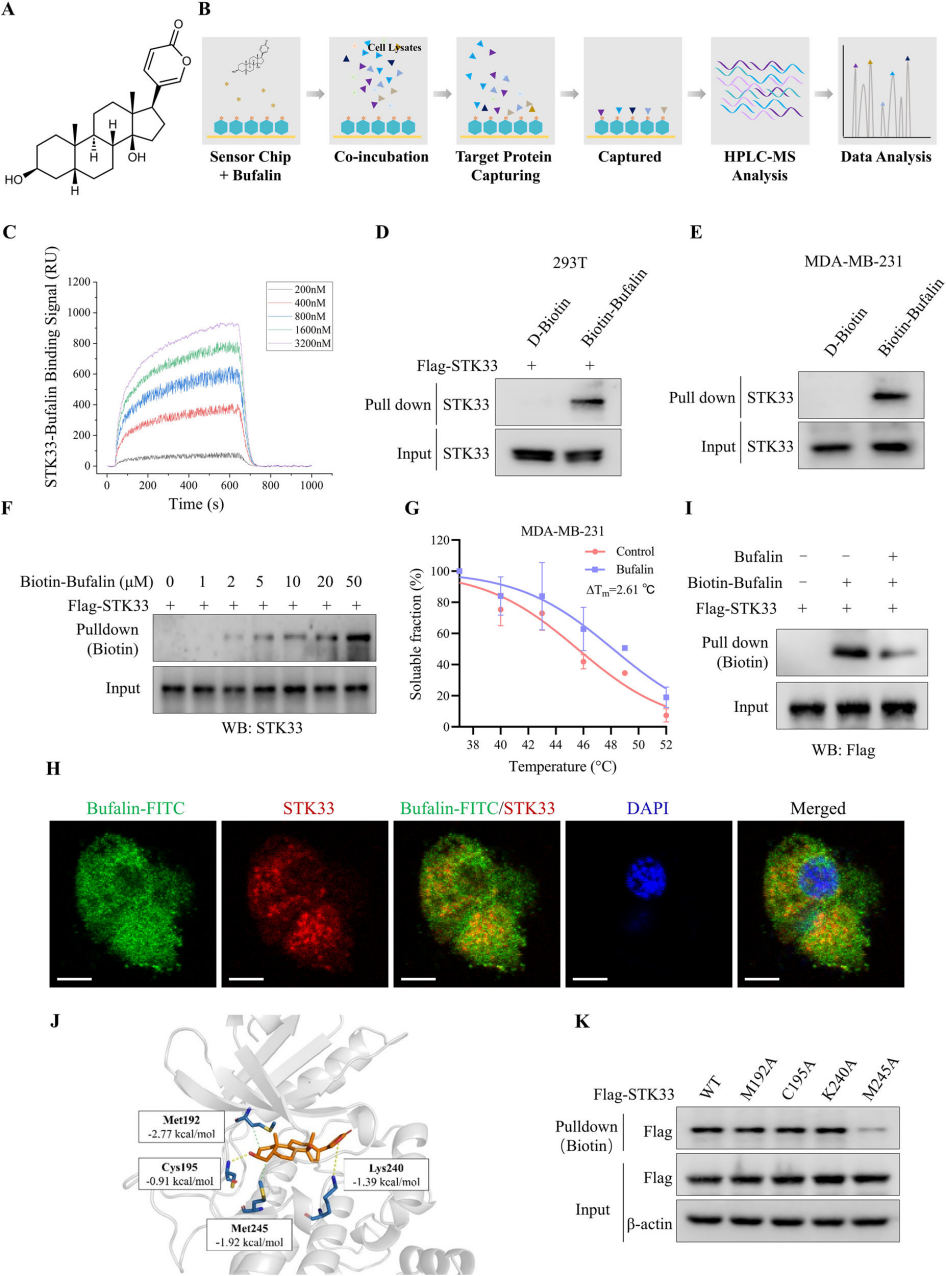
Identification of STK33 as a binding protein of Bufalin. A) The structure of Bufalin. B) The schematic generation of the SPR‐LC‐MS/MS approach. Bufalin is immobilized on a sensor chip and incubated with the cell lysates, followed by SPR analysis. The potential targets of Bufalin from Bufalin‐protein mixtures were screened and ranked by mass spectrometry analysis. C) SPR graph showing the interaction of Bufalin and STK33 recombinant protein. D) 293T cell was transfected with Flag‐STK33 plasmid, after transfected 48h, the cell lysates were incubated with D‐Biotin or Biotin‐Bufalin at 4 °C overnight, followed by pull‐down with streptavidin magnetic beads. The proteins bound to the magnetic beads were separated by SDS‐PAGE, followed by western blot using the STK33 antibody. E) The MDA‐MB‐231 cell lysates were incubated with D‐Biotin or Biotin‐Bufalin at 4 °C overnight, followed by pull‐down with streptavidin magnetic beads. The proteins bound to the magnetic beads were separated by SDS‐PAGE, followed by western blot using the STK33 antibody. F) 293T cell was transfected with Flag‐STK33 plasmid, after transfected 48h, the cell lysates were incubated with a series of concentrations of Biotin‐Bufalin at 4 °C overnight, followed by pull‐down with streptavidin magnetic beads. The proteins bound to the magnetic beads were separated by SDS‐PAGE, followed by western blot using the STK33 antibody. G) The thermal shift assay experiment was used to evaluate the binding interaction between Bufalin and STK33. H) The cellular location of STK33 and Biotin‐Bufalin was examined by immunofluorescence staining in MDA‐MB‐231 cells (Scale bar: 5 µm). I) 293T cell was transfected with Flag‐STK33 plasmid, after transfected 48h, the cells were incubated with Bufalin for 4h, then the cell lysates were incubated with Biotin‐Bufalin at 4 °C, followed by pull‐down with streptavidin magnetic beads. The proteins bound to the magnetic beads were separated by SDS‐PAGE, followed by western blot using the Flag antibody. J) The representative conformation from molecular dynamics (MD) simulations of the STK33‐Bufalin complex. K) 293T cell was transfected with the STK33 WT plasmid or mutant plasmid. After transfected 48h, the cell lysates were incubated with Biotin‐Bufalin at 4 °C overnight, followed by pull‐down with streptavidin magnetic beads. The proteins bound to the magnetic beads were separated by SDS‐PAGE, followed by western blot using the Flag antibody.

To clarify the molecular mechanism by which Bufalin (**Figure** **1**A) inhibits cancer growth, we performed the SPR‐LC‐MS/MS approach to screen the targets of Bufalin. Bufalin was immobilized on a sensor chip and incubated with MDA‐MB‐231 cell lysates, followed by SPR analysis. The potential targets of Bufalin from Bufalin‐protein mixtures were detected by mass spectrometry analysis (Figure 1B). Fifty‐one candidate proteins were identified, and we selected three top‐ranked, tumor‐related candidates (STK33, CLCN3, and RhoA) from the top 15 ranked proteins for further validation using SPR assays (Figure S1A, Supporting Information). Data presented in Figures 1C and S1B (Supporting Information) revealed strong binding affinities between recombinant STK33 and CLCN3 proteins with Bufalin (Table S1, Supporting Information). To further confirm these interactions, we synthesized biotinylated Bufalin conjugates and performed pull‐down assays. It was observed that Biotin‐Bufalin specifically pulled down STK33 in a dose‐dependent manner (Figure 1D–F), whereas no interaction was detected with CLCN3 (Figure S1C, Supporting Information). Moreover, Bufalin treatment increased the thermal stability of STK33 (Figure 1G), and the Biotin‐Bufalin and STK33 were co‐localized in the TNBC cells (Figure 1H). To clarify whether Bufalin could target STK33 in living cells, Bufalin was added to 293T cells with Flag‐STK33, then the cell lysates were incubated with Biotin‐Bufalin at 4 °C, followed by pull‐down assays with streptavidin magnetic beads. The results indicated that the addition of native Bufalin induced a competitive binding effect, as shown in Figure 1I. These data suggest that Bufalin interacts with STK33.

To elucidate the binding mechanism between Bufalin and STK33, we first identified potential interaction sites through computational analysis. Energy decomposition using the MM‐PBSA method revealed four key STK33 residues (M192, C195, K240, and M245) that likely contribute to Bufalin binding. Subsequent structural analysis of representative conformations from molecular dynamics simulations showed distinct interaction patterns: C195 and K240 primarily form hydrogen bonds with Bufalin, while M192 and M245 engaged mainly in hydrophobic interactions (Figure 1J). Notably, functional validation experiments demonstrated that among these residues, only the M245A mutation abolished STK33's binding capacity to Biotin‐Bufalin, whereas other mutants retained binding affinities comparable to wild‐type STK33 (Figure 1K). These results establish M245 as the critical residue mediating the STK33‐Bufalin interaction.

### 2.2 High STK33 Expression Correlates with Poor Therapeutic Outcome and Facilitates the Proliferation and Migration of TNBC Cells

**Figure 2:**
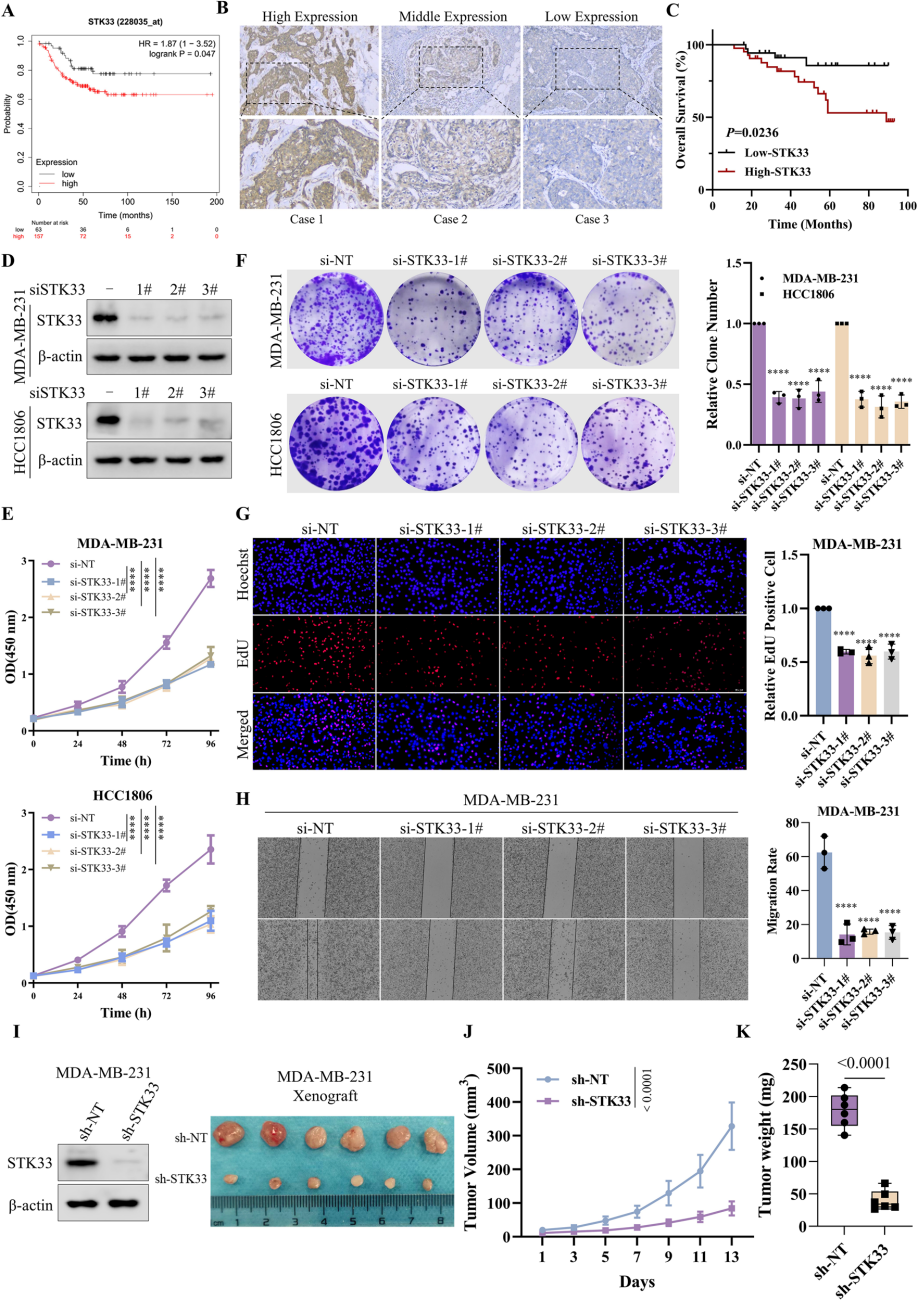
High STK33 expression correlates with poor therapeutic outcome and facilitates the proliferation and migration of TNBC cells. A) Kaplan–Meier overall survival analysis of TNBC patients based on the STK33 expression levels from TCGA. B) Representative IHC image of STK33 in TNBC tissues. C) Kaplan–Meier plots of the overall survival based on STK33 expression in 79 TNBC patients. D) MDA‐MB‐231 and HCC1806 cells were transfected with STK33 siRNA and non‐targeting siRNA, and the expression of STK33 protein was measured by western blot. E) CCK‐8 assay was applied to examine the cell viability of MDA‐MB‐231 and HCC1806 cells after silencing STK33; the data are presented as mean ± SD of three independent experiments. One‐way ANOVA was used to determine statistical significance, ^****^ *P* < 0.0001. F) MDA‐MB‐231 and HCC1806 cells were transfected with STK33 siRNA and non‐targeting siRNA, and cell proliferation was measured by the colony formation assay; the data are presented as mean ± SD of three independent experiments. One‐way ANOVA was used to determine statistical significance, ^****^ *P* < 0.0001. G) MDA‐MB‐231 cell was transfected with STK33 siRNA and non‐targeting siRNA, and cell proliferation was measured using the EdU kit; the data are presented as mean ± SD of three independent experiments. One‐way ANOVA was used to determine statistical significance, ^****^ *P* < 0.0001. H) Wound healing assays were performed in STK33‐silenced MDA‐MB‐231 cells and their corresponding negative control cells; the data are presented as mean ± SD of three independent experiments. One‐way ANOVA was used to determine statistical significance, ^****^ *p* < 0.0001. I–K) 4‐week‐old female nude mice were inoculated with MDA‐MB‐231 sh‐STK33 or MDA‐MB‐231 sh‐NT cells. Subcutaneous tumors were excised and photographed at the end of the experiment (I). Tumor sizes were measured on the specified days (J). Tumor weights were measured at the end of the experiments (K). The data represent the mean ± SD of 6 mice in each group; an unpaired t‐test was used to determine statistical significance, and *P* < 0.05 was considered to be statistically significant.

Analysis of the TCGA database uncovered that elevated STK33 expression was associated with poor prognosis of patients with TNBC patients (**Figure** **2**A), and tissue microarray data from the human protein atlas database showed that the STK33 expression was higher in breast cancer tissue as compared to normal breast tissue (Figure S2A, Supporting Information), and was markedly increased in TNBC tumors compared to levels in non‐TNBC tumors (Figure S2B, Supporting Information). Moreover, results of immunohistochemistry (IHC) staining assay confirmed that the percentage of high expression of STK33 was 53.16% in TNBC specimens we collected (Figure 2B; Table S2, Supporting Information), and decreased STK33 expression exhibited a positive correlation with prolonged survival in TNBC patients (Figure 2C). STK33 expression analysis revealed markedly higher levels in TNBC cell lines (MDA‐MB‐231 and HCC1806) compared to ER (+) (MCF‐7 and T47D) and HER2 (+) (SK‐BR‐3 and JIMT1) breast cancer cell lines, where expression was nearly undetectable (Figure S2C, Supporting Information). Silencing of STK33 expression resulted in suppression of TNBC cell proliferation, as evidenced by decreased cell viability, colony formation, and the number of EdU‐positive cells (Figure 2D–G). It also significantly inhibited cell migration as confirmed by results from the wound healing and trans‐well assays (Figures 2H; S2D,E, Supporting Information). In vivo experiments based on a subcutaneous xenograft model revealed that the volume and weight of tumors were significantly reduced in the nude mice bearing the STK33 shRNA tumors relative to those of mice bearing the control tumors (Figure 2I–K). Collectively, these results suggested that the STK33 was overexpressed in TNBC and high STK33 expression facilitates the proliferation and migration of TNBC cells, thereby predicting poor therapeutic outcomes.

### 2.3 STK33 Promotes TNBC Cell Proliferation by Increasing the Protein Stability of CCAR1

**Figure 3:**
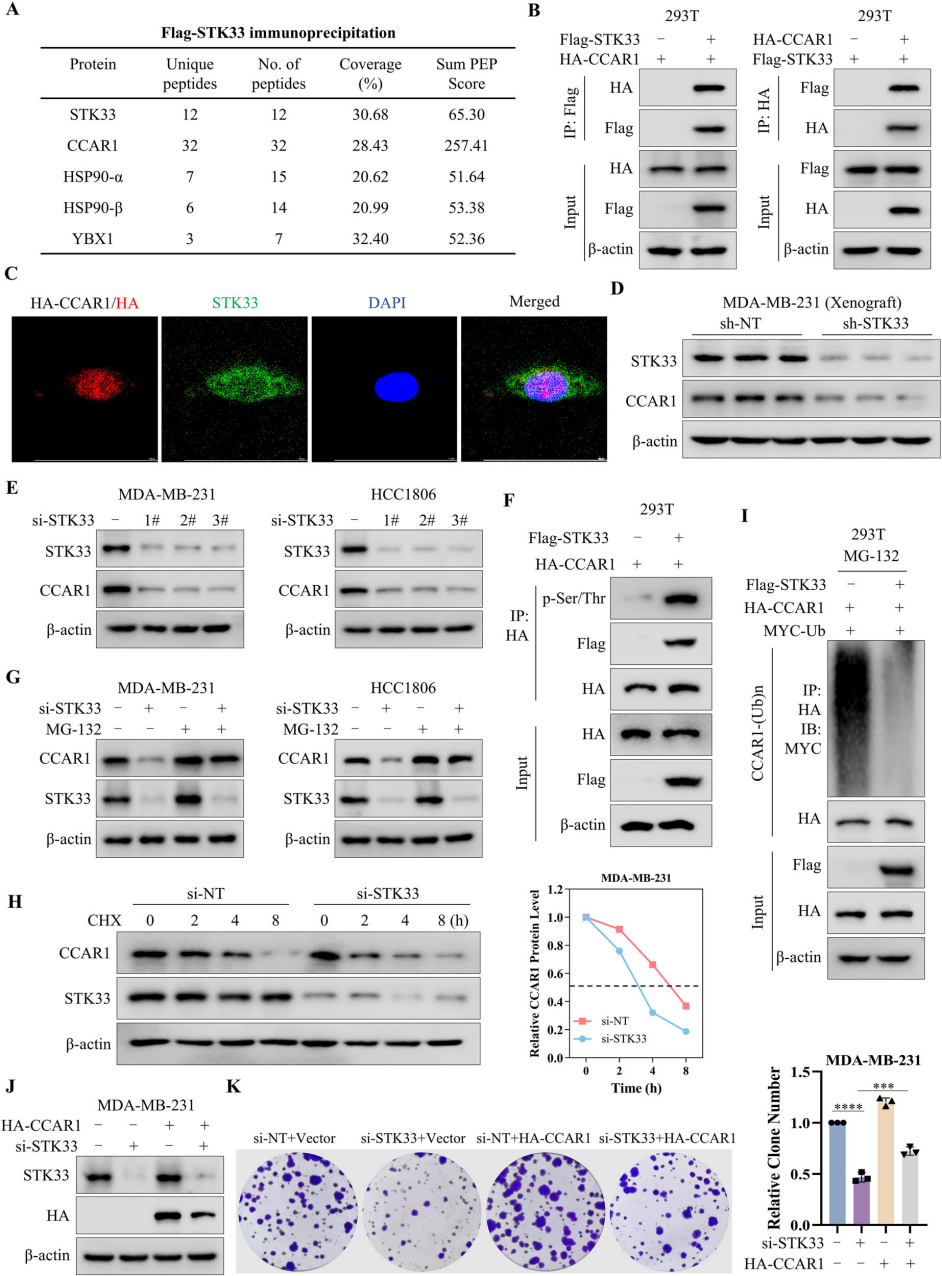
STK33 promotes TNBC cell proliferation by increasing the protein stability of CCAR1. A) The proteins immunoprecipitated by anti‐Flag antibody were analyzed by mass spectrometry, and the number of peptides for each protein identified was listed. B) 293T cells were transfected with Flag‐STK33 and HA‐CCAR1 plasmids, and then subjected to immunoprecipitation with anti‐Flag or anti‐HA antibodies. The lysates and immunoprecipitates were then blotted. C) MDA‐MB‐231 cells were transfected with an HA‐CCAR1 plasmid, and immunofluorescence staining was performed to detect the localization of HA (red) and STK33 (green) within the cells. D) The expression of STK33 and CCAR1 in MDA‐MB‐231 xenograft were measured by western blot. E) MDA‐MB‐231 and HCC1806 cells were transfected with STK33 siRNA or non‐targeting siRNA, and the expression of STK33 and CCAR1 was measured by western blot. F) 293T cells were transfected with Flag‐STK33 and HA‐CCAR1, and then subjected to immunoprecipitation with anti‐HA antibody, and the phosphorylation of CCAR1 was measured by western blot using p‐Ser/Thr antibody. G) MDA‐MB‐231 and HCC1806 cells were transfected with STK33 siRNA, followed by treatment with 10 µm MG132 for 4h before harvest. The expression of STK33 and CCAR1 was measured by western blot. H) MDA‐MB‐231 cells were transfected with STK33 siRNA or non‐targeting siRNA, and followed treated with 10µg mL^−1^ of cycloheximide (CHX) and harvested at the indicated time points. The protein levels of CCAR1 and STK33 were detected by western blot. I) 293T cells were transfected with HA‐CCAR1, MYC‐Ub, or Flag‐STK33 plasmids, followed by treatment with MG132 (10 µm) for 10 h before harvest. Then the cell lysates were subjected to immunoprecipitation with anti‐HA antibody and blotted with anti‐MYC antibody. J) MDA‐MB‐231 cells were transfected with STK33 siRNA, and then transfected with HA‐CCAR1, the expression of STK33 and HA was measured by western blot. K) MDA‐MB‐231 cells were transfected with STK33 siRNA, and then transfected with HA‐CCAR1, and cell proliferation was measured by the colony formation assay. The data are presented as mean ± SD of three independent experiments. One‐way ANOVA was used to determine statistical significance, ^***^ *P* < 0.001, ^****^ *P* < 0.0001.

To investigate the mechanism by which STK33 promotes cell proliferation, we conducted mass spectrometry (MS) to identify STK33‐binding proteins in MDA‐MB‐231 cells. The results indicated that cell division cycle and apoptosis regulator protein 1 (CCAR1) exhibited a strong binding affinity for STK33 in **Figure** **3**A. Researchers have uncovered that STK33 is a member of the CaMKs, and most of their substrates contain a conserved Arg‐NB‐X‐Ser/Thr‐HP (R‐NB‐X‐S/THP) motif.^[^ ^27^, ^28^ ^]^ Three serine sites (329, 333, and 343) were identified in the CCAR1 containing the R‐NB‐X‐S/T‐HP motif (Figure S3A, Supporting Information), and the Ser343 site was responsible for CCAR1 stability.^[^ ^29^ ^]^ Co‐immunoprecipitation and immunofluorescence assays validated the interaction between STK33 and CCAR1 (Figure 3B,C). STK33 silencing caused a significant decrease in CCAR1 protein expression (Figure 3D,E), while exogenously expressed STK33 led to upregulation of CCAR1 in TNBC cells (Figure S3B, Supporting Information). A positive correlation was recorded between STK33 and CCAR1 in basal‐like breast cancer (Figure S3C, Supporting Information). We also demonstrated that STK33 increased the phosphorylation of CCAR1 (Figure 3F). To further investigate how STK33 regulates CCAR1 stability, we combined STK33 knockdown with proteasome inhibition using MG‐132. The results showed that MG‐132 rescued the STK33 knockdown‐induced downregulation of CCAR1 protein expression (Figure 3G). Similarly, inhibition of ubiquitin activation using MLN4924, an E1 ubiquitin‐activating enzyme inhibitor, also prevented the reduction of CCAR1 expression caused by STK33 knockdown (Figure S3D, Supporting Information). Furthermore, the CHX assay demonstrated that STK33 knockdown significantly shortened the half‐life of CCAR1 protein (Figure 3H). To further elucidate the mechanism, we found that STK33 overexpression inhibited CCAR1 ubiquitination (Figure 3I), whereas STK33 silencing significantly enhanced CCAR1 ubiquitination (Figure S3E, Supporting Information). Gene Set Enrichment Analysis (GSEA) revealed a positive correlation between STK33 expression and Wnt/β‐catenin signaling, a downstream pathway of CCAR1 (Figure S3F, Supporting Information). Silencing of STK33 led to a significant decrease in β‐catenin protein levels (Figure S3G, Supporting Information). In addition, overexpressed of CCAR1 rescued the inhibition of cell proliferation induced by STK33 knockdown (Figures 3J,K; S3H,I, Supporting Information). These results suggest that STK33 increases CCAR1 stability by phosphorylation to promote TNBC cell proliferation.

**Figure 4:**
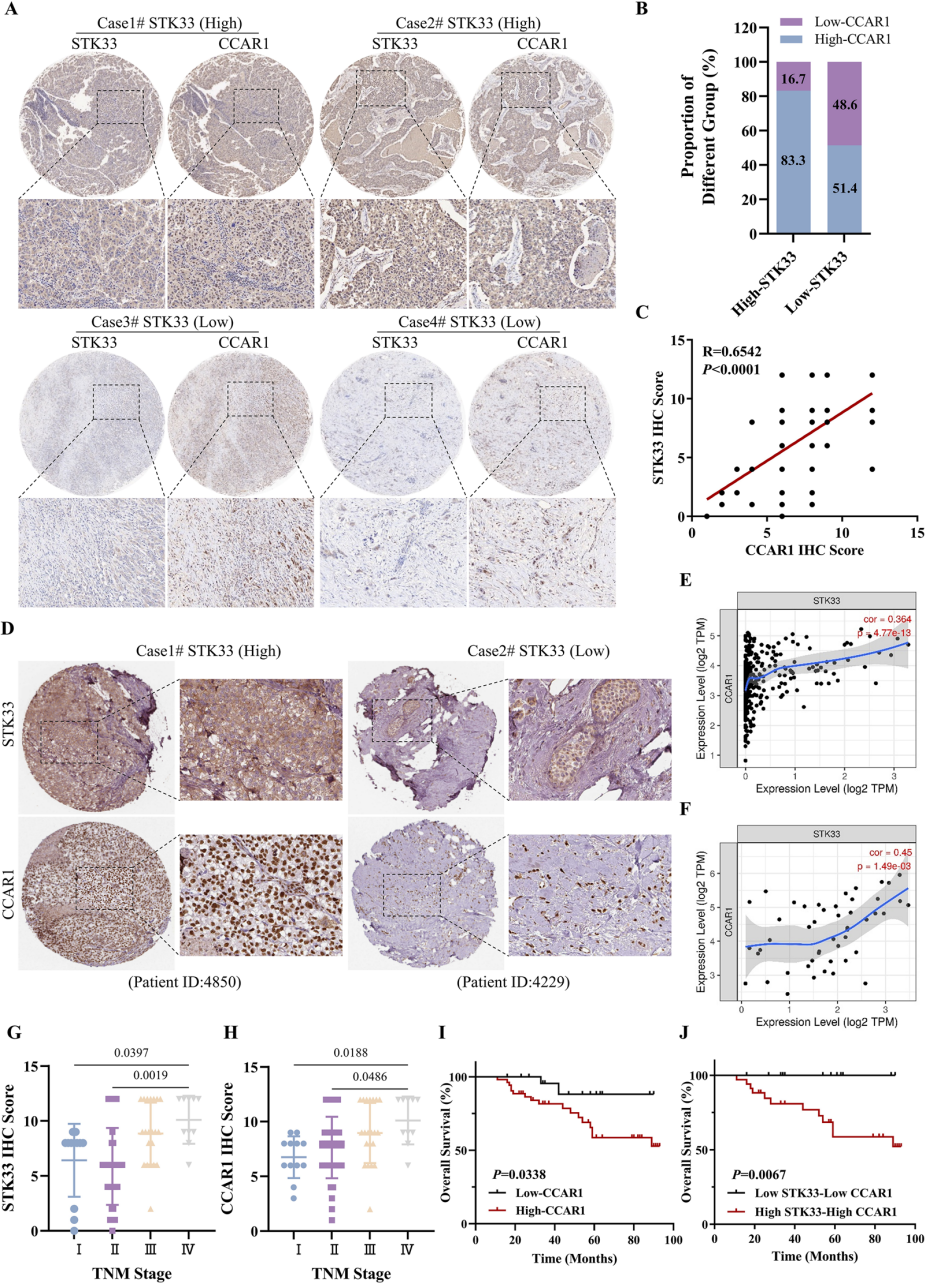
High expression of the STK33‐CCAR1 axis is correlated with poor survival in patients with TNBC. A) Representative IHC staining for STK33 and CCAR1 in TNBC. Cases 1 and 2 are representative of a patient with STK33‐high TNBC. Cases 3 and 4 are representative of a patient with STK33‐low TNBC. B, C) Pearson's correlation analyses of STK33 and CCAR1. D) IHC analyses of STK33 and CCAR1 levels in breast cancer tissues from the Human Protein Atlas database (https://www.proteinatlas.org/). E) Pearson's correlation analyses of STK33 and CCAR1 mRNA levels in liver hepatocellular carcinoma from the TIMER web server (https://cistrome.shinyapps.io/timer/). F) Pearson's correlation analyses of STK33 and CCAR1 mRNA levels in diffuse large B‐cell lymphoma from the TIMER web server (https://cistrome.shinyapps.io/timer/). G) The correlation between STK33 expression and the histology grade in TNBC patients, the data are presented as mean ± SD, one‐way ANOVA was used to determine statistical significance, *P* < 0.05 was considered to be statistically significant. H) The correlation between CCAR1 expression and the histology grade in TNBC patients, the data are presented as mean ± SD, one‐way ANOVA was used to determine statistical significance, *P* < 0.05 was considered to be statistically significant. I) Kaplan–Meier plots of the overall survival based on CCAR1 expression in TNBC patients. J) Kaplan–Meier curves of overall survival based on STK33 and CCAR1 expression in TNBC patients.

To confirm the relationship between STK33 and CCAR1, we performed IHC staining on the TNBC tissue microarray. As shown in **Figure** **4**A–C, expression of STK33 had a significant positive correlation with expression of CCAR1. This finding was validated on the Human Protein Atlas (https://www.proteinatlas.org/) (Figure 4D). In addition, the strong positive correlation between STK33 and CCAR1 expression was also observed in liver hepatocellular carcinoma and diffuse large B‐cell lymphoma (Figure 4E,F). Furthermore, the expression of STK33 or CCAR1 was positively associated with the TNM stage (Figure 4G,H), and the high expression of STK33 and CCAR1 is associated with shorter survival of TNBC patients based on the Kaplan–Meier analysis (Figure 4I,J). Collectively, these data indicated that the STK33‐CCAR1 axis was increased in TNBC and can be a molecular marker for predicting the prognosis of TNBC.

### 2.4 Bufalin Causes the Proteasomal Degradation of STK33 by Destroying the STK33‐HSP90 Complex Formation

**Figure 5:**
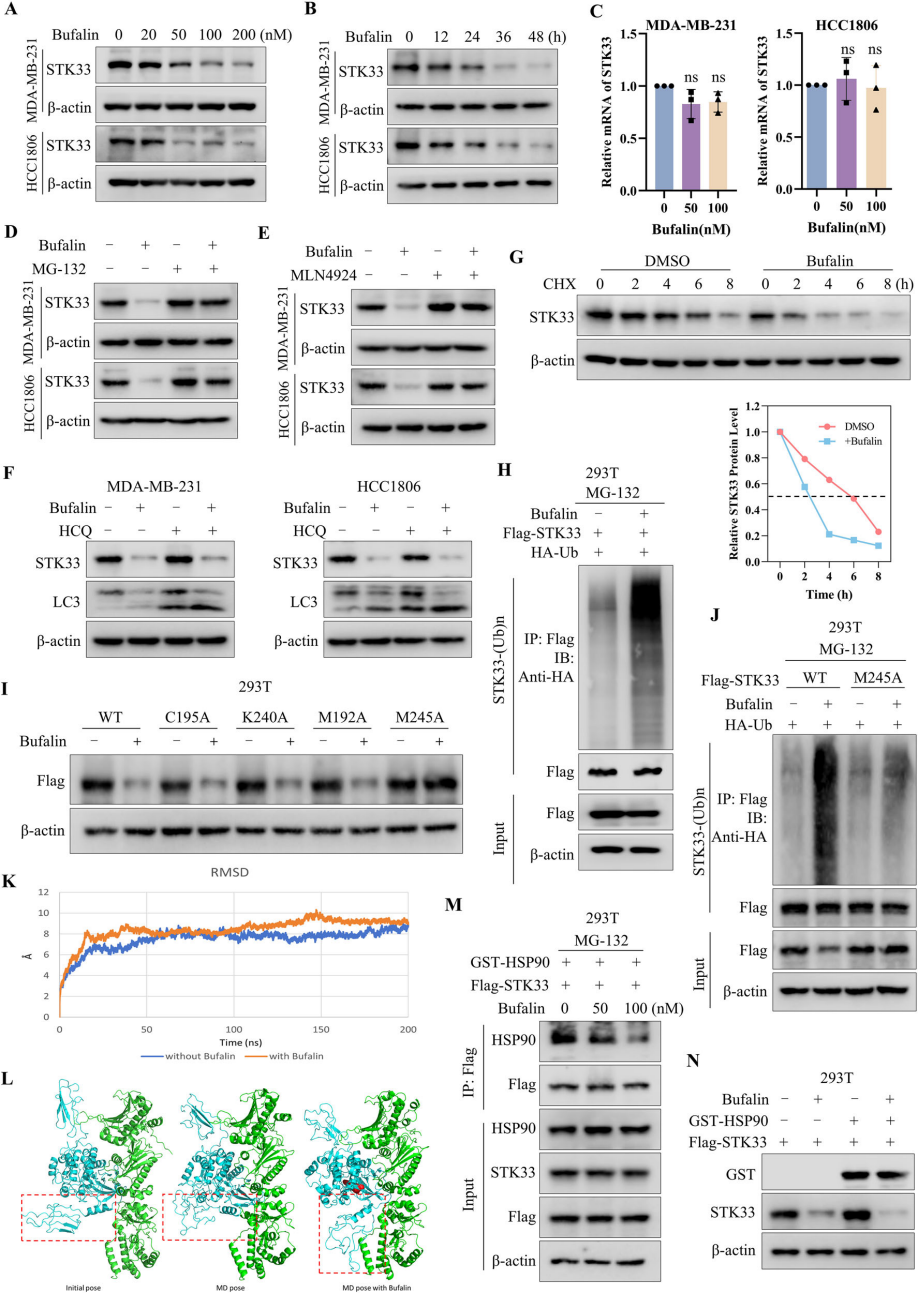
Bufalin causes the proteasomal degradation of STK33 by destroying the STK33‐HSP90 complex formation. A) MDA‐MB‐231 and HCC1806 cells were treated with a series of concentrations of Bufalin for 48 h, and STK33 levels were measured by western blot. B) MDA‐MB‐231 and HCC1806 cells were treated with Bufalin for different periods of time, and STK33 levels were measured by western blot. C) MDA‐MB‐231 and HCC1806 cells were treated with a series of concentrations of Bufalin for 48 h; the STK33 mRNA level was analyzed by real‐time PCR, and the data are presented as mean ± SD of three independent experiments. One‐way ANOVA was used to determine statistical significance, ns, *P* > 0.05. D) MDA‐MB‐231 and HCC1806 cells were treated with Bufalin for 48 h, followed by treatment with 10 µm MG132 for 4h. The expression of STK33 was measured by western blot. E) MDA‐MB‐231 and HCC1806 cells were treated with Bufalin for 48 h in the presence or absence of MLN4924. The expression of STK33 was measured by western blot. F) MDA‐MB‐231 and HCC1806 cells were treated with Bufalin in the presence or absence of HCQ. The expression of STK33 and LC3 was measured by western blot. G) MDA‐MB‐231 cells were treated with Bufalin, and followed treated with 10µg mL^−1^ of cycloheximide (CHX) and harvested at the indicated time points. The expression of STK33 was measured by western blot. H) 293T cells were transfected with Flag‐STK33 and HA‐Ub plasmid, and then subjected to Bufalin for 48 h, followed by treatment with MG132 (10 µm) for 10 h before harvest. Then the cell lysates were subjected to immunoprecipitation with anti‐Flag antibodies and blotted with anti‐HA antibodies. I) 293T cells were transfected with STK33 WT or mutant plasmid, followed by treatment with Bufalin for 48 h. The expression of Flag was measured by western blot. J) 293T cells were transfected with Flag‐STK33 WT or mutant plasmid and HA‐Ub plasmid, and then subjected to Bufalin for 48 h, followed by treatment with MG132 (10 µm) for 10 h before harvest. Then the cell lysates were subjected to immunoprecipitation with anti‐Flag antibodies and blotted with anti‐HA antibodies. K) RMSD plots of the STK33‐HSP90 complex with or without Bufalin. L) The binding poses of STK33 and HSP90 with or without Bufalin. M) 293T cells transfected with Flag‐STK33 and GST‐HSP90 plasmid were treated with Bufalin at the indicated concentration, then lysed, and lysates were subjected to immunoprecipitation with anti‐Flag antibodies. Proteins retained on Sepharose were blotted with the indicated antibodies. N) 293T cells were transfected with Flag‐STK33 and GST‐HSP90 plasmids, followed by treatment with Bufalin for 48 h. The STK33 and GST protein levels were measured by western blot.

Next, we explored the effect of Bufalin on STK33 protein turnover. As shown in **Figure** **5**A,B, Bufalin treatment caused a dose‐ and time‐dependent decrease in STK33 expression, but it did not alter the mRNA level of STK33 in TNBC cells (Figure 5C). Treatment with MG132 and MLN4924 reversed the down‐regulation of STK33 in cells treated with Bufalin (Figure 5D,E). Moreover, the lysosomal inhibitor Hydroxychloroquine (HCQ) did not induce any rescue effects (Figure 5F). The CHX chase assay showed that Bufalin treatment significantly promoted the STK33 turnover and shortened its half‐life (Figure 5G). Bufalin treatment significantly increased the ubiquitination of STK33 protein (Figures 5H; S4A, Supporting Information). In addition, the expression of CCAR1 was also downregulated after treatment with Bufalin (Figure S4B, Supporting Information). Consistent with the previous results, mutation of the M245 site abolished Bufalin‐induced STK33 downregulation and ubiquitination (Figure 5I,J). The results suggest that Bufalin has a role in promoting the degradation of STK33.

Researchers have revealed that HSP90 binds and stabilizes STK33.^[^ ^26^ ^]^ Here, we indicated that HSP90 overexpression significantly increased the expression of STK33, and depletion of HSP90 by siRNA markedly reduced the levels of STK33 protein (Figure S4C–E, Supporting Information). Next, we investigated whether Bufalin alters the binding of STK33 with HSP90. Notably, the RMSD trend of STK33 and HSP90 was enhanced following Bufalin treatment as confirmed by the results of the molecular dynamics simulation analysis (Figure 5K). Further, the binding free energy between HSP90 and STK33 was increased from −30.6261 to −21.6760 kcal mol^−1^ in the presence of Bufalin (Figure 5L; Table S3, Supporting Information). These results suggested that Bufalin inhibited the binding of STK33 with HSP90. Moreover, immunoprecipitation assays revealed that increasing concentrations of Bufalin progressively weakened the interaction between HSP90 and STK33 (Figure 5M). Furthermore, Bufalin had no significant effect on STK33 expression when HSP90 was knockdown (Figure S4F, Supporting Information), whereas ectopic expression of HSP90 lost the ability to upregulate STK33 in the presence of Bufalin (Figures 5N; S4G, Supporting Information). Together, our results provide evidence that Bufalin promotes the degradation of STK33 by inhibiting the binding of STK33 with HSP90.

### 2.5 Cytotoxicity of Bufalin is Associated with Down‐Regulating STK33 in TNBC

**Figure 6:**
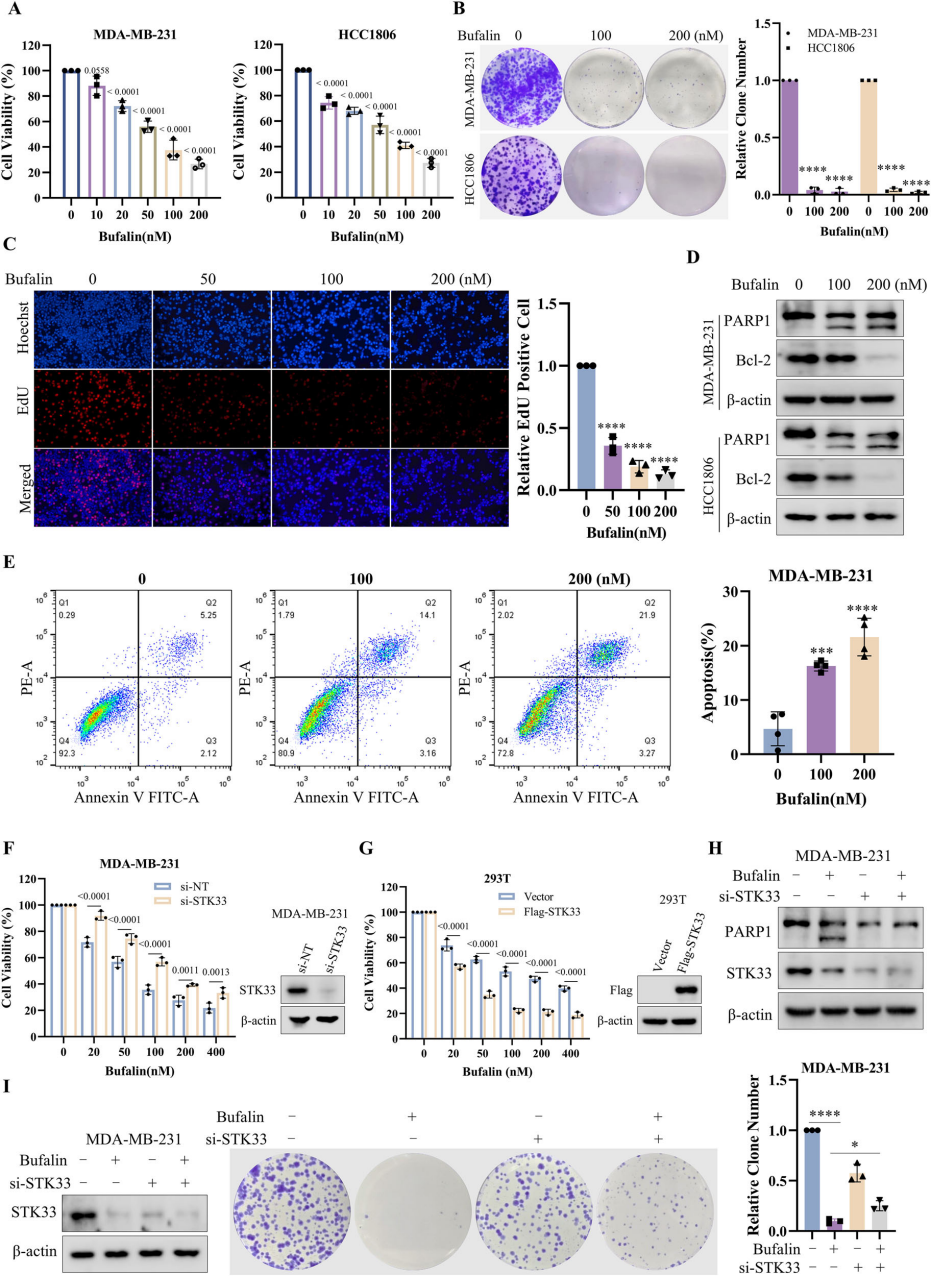
Cytotoxicity of Bufalin is associated with down‐regulating STK33 in TNBC. A) MDA‐MB‐231 and HCC1806 cells were treated with Bufalin, and cell viability was determined using the CCK‐8 assay; the data are presented as mean ± SD of three independent experiments. One‐way ANOVA was used to determine statistical significance; *P* < 0.05 was considered to be statistically significant. B) The colony formation assay was used to measure cell proliferation in MDA‐MB‐231 and HCC1806 cells after treatment with Bufalin; the data are presented as mean ± SD of three independent experiments. One‐way ANOVA was used to determine statistical significance, ^****^ *P* < 0.0001. C) The EdU assay was used to measure cell proliferation in MDA‐MB‐231 cells following treatment with Bufalin; the data are presented as mean ± SD of three independent experiments. One‐way ANOVA was used to determine statistical significance, ^****^ *P* < 0.0001. D) MDA‐MB‐231 and HCC1806 cells were treated with 100 or 200 nm Bufalin for 48 h, and the expressions of PARP1 and Bcl‐2 were measured by western blot. E) MDA‐MB‐231 cells were treated with 100 or 200 nm Bufalin for 48 h, and the apoptosis was examined by measuring Annexin V staining. The data are presented as mean ± SD of four independent experiments. One‐way ANOVA was used to determine statistical significance, ^***^ *P* < 0.001, ^****^ *P* < 0.0001. F) MDA‐MB‐231 cells were transfected with STK33 siRNA, followed by treatment with Bufalin for 72 h, and the cell viability was determined using the CCK‐8 assay. The data are presented as mean ± SD of three independent experiments. Two‐way ANOVA was used to determine statistical significance; *P* < 0.05 was considered to be statistically significant. G) 293T cells were transfected with Flag‐STK33 plasmid, followed by treatment with Bufalin, and the cell viability was determined using the CCK‐8 assay; the data are presented as mean ± SD of three independent experiments. Two‐way ANOVA was used to determine statistical significance; *P* < 0.05 was considered to be statistically significant. H) MDA‐MB‐231 cells were transfected with STK33 siRNA, followed by treatment with Bufalin for 48 h, and the PARP1 and STK33 were measured by western blot. I) MDA‐MB‐231 cells were transfected with STK33 siRNA, followed by treatment with Bufalin, and the colony‐formation assay was measured. The data are presented as mean ± SD of three independent experiments. One‐way ANOVA was used to determine statistical significance, ^*^ *P* <0.05, ^****^ *P* < 0.0001.

To further investigate the link between Bufalin's anti‐cancer effects and STK33 expression in TNBC, the influence of Bufalin on cell proliferation and apoptosis was explored. As shown in **Figure** **6**A, Bufalin exhibited a concentration‐dependent inhibition of cell viability and significantly inhibited cell proliferation (Figure 6B) and reduced the EdU‐positive cell number of TNBC cells (Figure 6C). Further, increased expression of cleaved‐PARP1 and decreased expression level of Bcl‐2 were detected in the tumor cells exposed to Bufalin (Figure 6D). Bufalin treatment induced apoptosis in a dose‐dependent manner, as evidenced by an increase in annexin V staining (Figure 6E). Moreover, the results indicated that STK33 silencing reduced the cytotoxic effects of Bufalin on TNBC cells (Figure 6F), while its overexpression promoted cellular sensitivity to Bufalin (Figure 6G). Similarly, STK33 silencing strongly reduced the Bufalin‐induced cleaved‐PARP1 expression (Figure 6H), and results of the colony formation assays confirmed that the tumor cells became less sensitive to Bufalin following STK33 knockdown (Figure 6I). These findings indicated that the cytotoxic effects of Bufalin were due to its inhibition of STK33.

### 2.6 Bufalin Exerts Anti‐Cancer Activity in Animal TNBC Model and In Patient‐Derived TNBC Organoids

**Figure 7:**
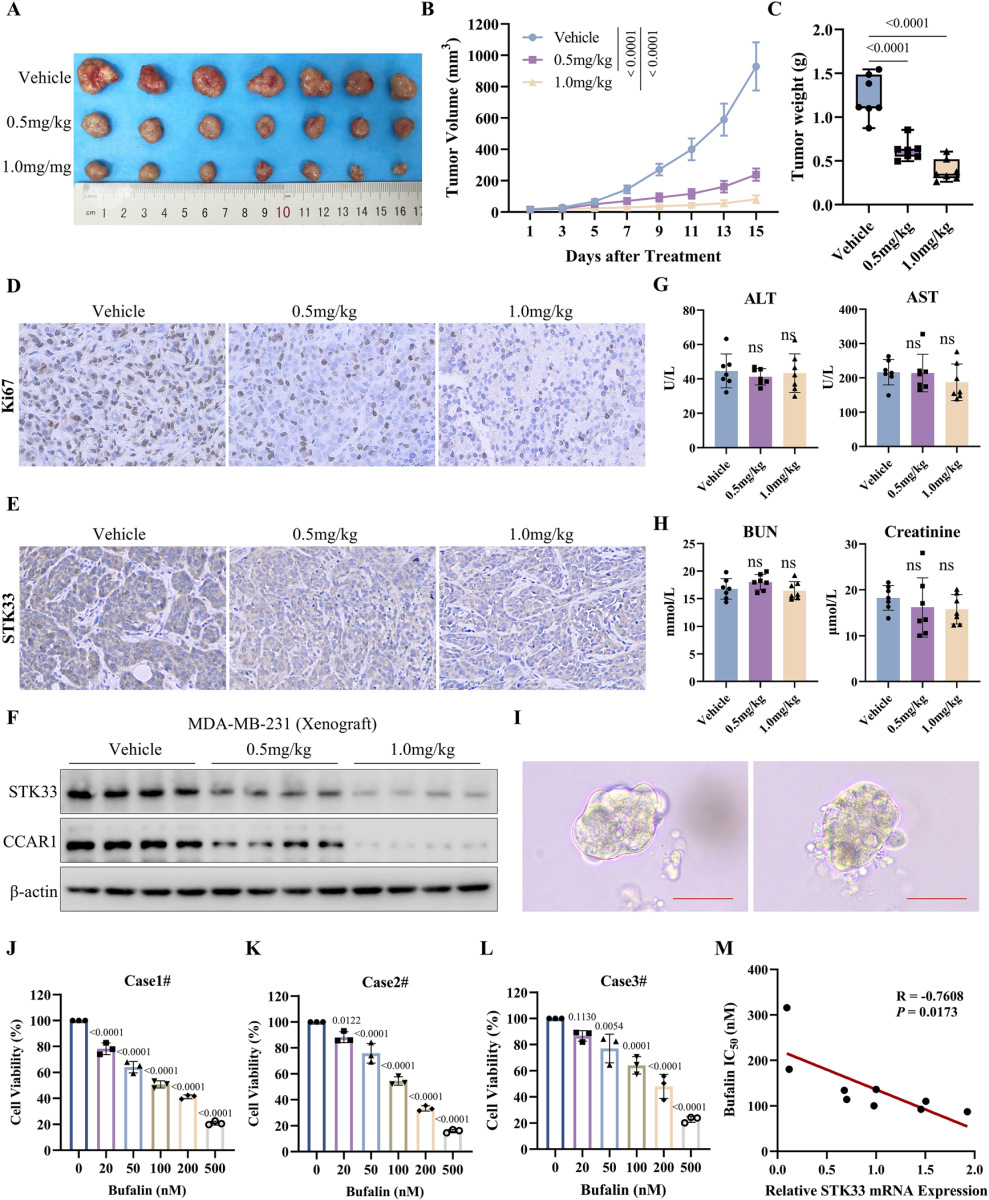
Bufalin exerts anti‐cancer activity in animal TNBC model and in patient‐derived TNBC organoids. 4‐week‐old female nude mice were inoculated with MDA‐MB‐231 cells. The tumor‐bearing mice were subsequently given the indicated treatment. A) Subcutaneous tumors were excised and photographed at the end of the experiment. B) Tumor sizes were measured on the specified days, and the data are presented as mean ± SD of 7 mice. One‐way ANOVA was used to determine statistical significance; *P* < 0.05 was considered to be statistically significant. C) Tumor weights were measured at the end of the experiments, and the data are presented as mean ± SD of 7 mice. One‐way ANOVA was used to determine statistical significance; *P* < 0.05 was considered to be statistically significant. D) Representative immunohistostaining images for detecting Ki67 expression in the tumor specimens. E) Representative immunohistostaining images for detecting STK33 expression in the tumor specimens. F) Western blot analysis of the STK33 and CCAR1 protein expression in xenograft tumors following the indicated treatment. G) Mice liver functions were measured at the end of the experiments, and the data are presented as mean ± SD of 7 mice. One‐way ANOVA was used to determine statistical significance, ns, *P* > 0.05. ALT, alanine aminotransferase; AST, aspartate aminotransferase. H) Mice kidney functions were measured at the end of the experiments, and the data are presented as mean ± SD of 7 mice. One‐way ANOVA was used to determine statistical significance, ns, *P* > 0.05. BUN, blood urea nitrogen. I) Representative images of TNBC patient‐derived organoids (Scale bar 50µm). J–L) The proliferation curve of TNBC PDOs treated with Bufalin, the data are presented as mean ± SD of three independent experiments. One‐way ANOVA was used to determine statistical significance; *P* < 0.05 was considered to be statistically significant. M) Spearman correlation analysis between STK33 expression and the IC_50_ of TNBC PDOs.

To demonstrate the antitumor activity of Bufalin in pre‐clinical settings, we quantified the therapeutic effects of Bufalin in a nude mouse xenograft model. As shown in **Figure** **7**A–C, Bufalin significantly prevented the growth of the tumor, as evidenced by significant reduction of tumor volumes and weights, as compared to the control group. Additionally, Ki67 expression was markedly reduced in Bufalin‐treated groups, indicating that Bufalin suppresses breast cancer cell proliferation in vivo (Figure 7D). We also examined the effect of Bufalin on the expression of STK33 in tumor tissues through immunohistochemistry and western blot assay. As shown in Figure 7E,F, Bufalin treatment significantly down‐regulated STK33 relative to the control. The concentration of Bufalin (0.5 and 1.0 mg kg^−1^) did not induce significant changes in hepatic function or renal function (Figure 7G,H).

To further validate the antitumor efficacy of Bufalin, we employed a patient‐derived TNBC organoid (PDO) model (Figure 7I). Bufalin treatment significantly suppressed the organoid growth, as demonstrated by quantitative growth inhibition assays (Figure 7J–L). Importantly, our results demonstrated a significant positive correlation between Bufalin sensitivity and STK33 expression in TNBC patient‐derived organoids (Figure 7M), suggesting STK33 expression may serve as a predictive biomarker for Bufalin response in TNBC.

## 3 Discussion

**Figure 8:**
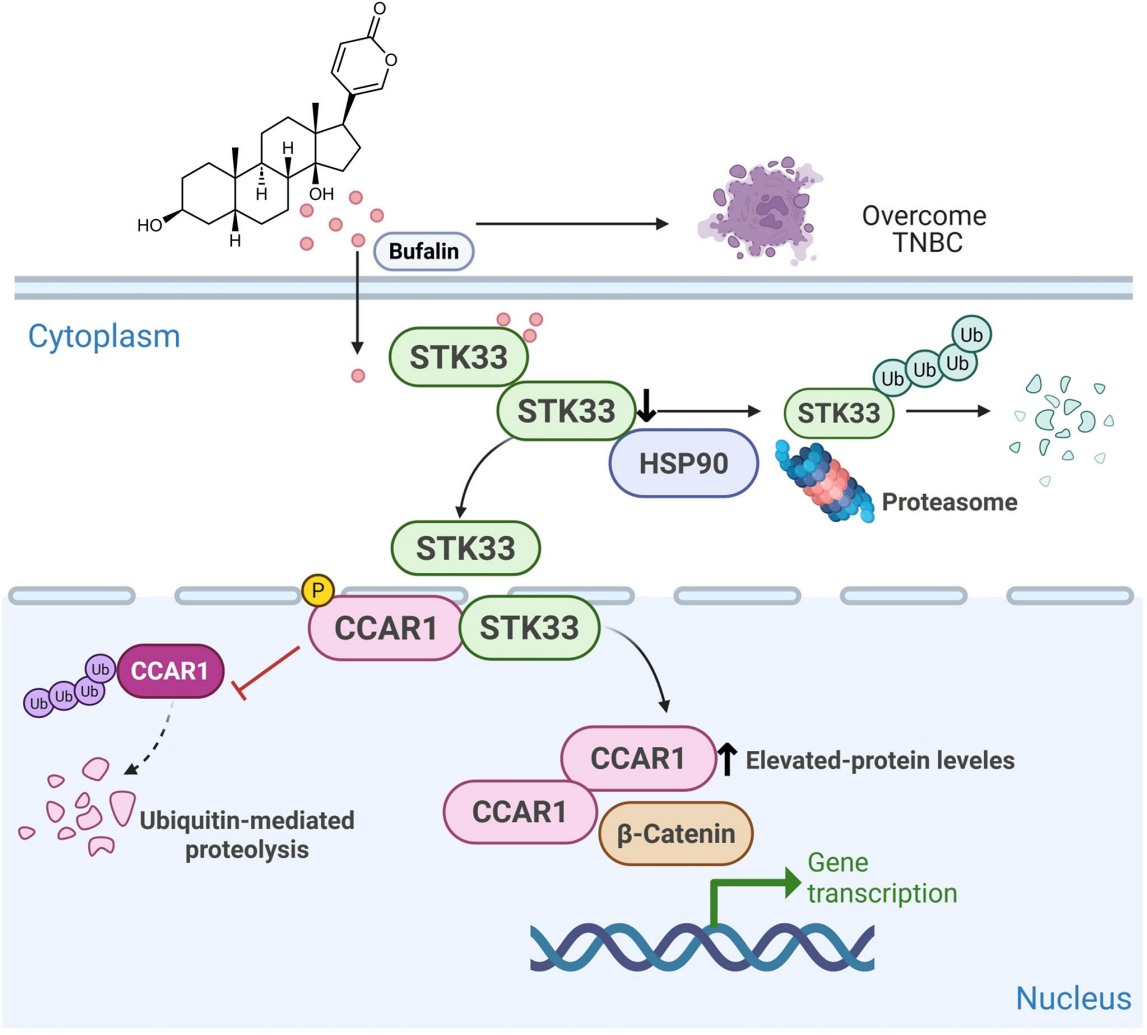
Regulatory signaling pathway of Bufalin in TNBC. In this study, we identified STK33 as a putative target of Bufalin. Bufalin disrupts the interaction between STK33 and HSP90, thereby promoting the ubiquitination and proteasomal degradation of STK33. Furthermore, STK33 is highly expressed in TNBC and enhances TNBC cell proliferation by phosphorylating and stabilizing CCAR1. Targeted degradation of STK33 by Bufalin significantly inhibits TNBC growth, highlighting its potential as a promising therapeutic candidate for TNBC treatment. Created in BioRender. Jiang, S. (2025) https://BioRender.com/00nde1g.

Bufalin (HuaChansu) was approved for cancer treatment, and several clinical investigations have recently demonstrated that it exerts anti‐cancer effects.^[^ ^8^ ^]^ Compelling evidence shows that Bufalin exerts antitumor effects by activating apoptosis, cell cycle arrest, and suppressing angiogenesis in diverse cancer cells.^[^ ^30^ ^]^ Here, we found that Bufalin inhibited TNBC by binding to STK33 and promoting its degradation. STK33, in turn, stimulates TNBC cell proliferation via phosphorylating CCAR1 and increasing its stability, which implies that STK33 is a potential therapeutic target for TNBC treatment (**Figure** **8**).

Evidence from recent investigations shows that Bufalin can promote protein degradation. A study by Liu et al found that Bufalin functioned as a molecular glue that enhanced E2F2 degradation and inhibited hepatocellular carcinoma growth in vitro and in vivo.^[^ ^31^ ^]^ Elsewhere, it was observed that Bufalin induced ferroptosis in non‐small cell lung cancer cells by promoting the ubiquitination and degradation of GPX4.^[^ ^32^ ^]^ In the present study, Bufalin treatment decreased STK33 levels in a time‐ and dose‐dependent manner in TNBC cells. We further revealed that Bufalin could weaken the HSP90‐STK33 complex formation, promote the ubiquitination of STK33, leading to its degradation.

Accumulating evidence confirms that elevated STK33 expression correlates with poor prognosis in cancer patients, making it a valuable marker for clinical diagnosis and prognosis.^[^ ^20^, ^33^ ^]^ Here, we demonstrate, for the first time, that STK33 is upregulated in TNBC and predicts poor prognosis. In addition, we indicate that STK33 knockdown significantly inhibits cell proliferation and migration, providing strong evidence that targeting STK33 may be an effective therapeutic approach for treating TNBC.

Currently, TNBC has limited treatment options. While PARP inhibitors (PARPi), including Olaparib and Talazoparib, have shown efficacy in BRCA‐mutated TNBC, their clinical application is frequently limited by acquired resistance.^[^ ^34^, ^35^ ^]^ Emerging evidence suggests that STK33 plays a crucial role in regulating DNA repair pathways,^[^ ^36^, ^37^, ^38^ ^]^ implying that STK33 inhibition could potentiate PARPi sensitivity by impairing homologous recombination (HR)‐mediated DNA repair. In addition, Olaparib has been identified as a substrate of the P‐gp transporter,^[^ ^39^ ^]^ suggesting that drug efflux may contribute to PARPi resistance. Notably, Bufalin has been demonstrated to reverse multidrug resistance (MDR) by down‐regulating P‐gp expression, thereby increasing intracellular drug accumulation.^[^ ^40^ ^]^ Together, these findings support the potential of combining Bufalin with PARPi as a promising therapeutic strategy to overcome PARPi resistance in TNBC.

Although STK33 was identified in 2001, few substrates of STK33 have been reported to date.^[^ ^27^ ^]^ In this study, we identified CCAR1 as an interacting protein of STK33 and revealed that STK33 phosphorylates CCAR1 to influence its stability. Moreover, the results indicated that elevated expression of STK33 and CCAR1 correlated with poor survival in breast cancer, suggesting that the STK33‐CCAR1 axis is likely to be a robust biomarker for predicting the prognosis of TNBC.

Researchers have uncovered that CCAR1 interacts with β‐catenin to increase its ability to activate target genes and regulate cell proliferation and apoptosis signaling.^[^ ^41^ ^]^ Here, we demonstrated that there is a positive correlation between STK33 expression and Wnt/β‐catenin signaling, and knockdown of STK33 dramatically decreases the protein level of β‐catenin. As the aberrant activation of Wnt/β‐catenin signaling is indispensable for maintaining cancer's malignant phenotype, our finding provides evidence that STK33 may be a key molecule in Wnt/β‐catenin signaling, and targeting STK33 is critical for the treatment of TNBC.

A growing body of evidence suggests that STK33 is a promising therapeutic target for cancer. However, small‐molecule inhibitors of STK33 identified so far have not shown any effects on cell viability.^[^ ^24^, ^42^ ^]^ Recent advancements in the design of targeted degradation of cancer‐promoting proteins, such as proteolysis targeting chimeric (PROTAC) technology, have opened a new avenue for establishing novel anticancer agents.^[^ ^43^ ^]^ Zhong et al proposed that targeting the degraders of STK33 is a valuable chemical tool for studying the role of STK33 in cancer cells.^[^ ^25^, ^26^ ^]^ These findings highlight the potential of targeting STK33 degradation as an anticancer therapeutic strategy. In this study, we report, for the first time, that the naturally produced Bufalin induces proteasome‐mediated degradation of STK33 and exhibits potent cytotoxic potency against TNBC. Our findings provide a complementary tool for developing STK33 degrader with potential anti‐tumor properties.

In summary, we demonstrate that STK33 may serve as a target of Bufalin in TNBC, which induces the degradation of STK33 and inhibits the growth of TNBC via targeting STK33. Notably, the STK33 expression was found to be correlated with poor prognosis in TNBC, which promotes cancer cell proliferation and migration via the CCAR1/β‐catenin pathway. Our study presents a promising therapeutic target and an anti‐tumor strategy for the management of TNBC.

## 4 Experimental Section

### Ethics Approval

The study protocol involving human participants was reviewed and approved by the Medical Ethics Review Committee of Xiangya Hospital, Central South University (Approval No. 2023121169). All human tissue samples were collected from Xiangya Hospital, Central South University (Changsha, China) with written informed consent provided by each participant prior to sample collection.

All animal experiments were conducted in compliance with institutional guidelines and were approved by the Institutional Animal Care and Use Committee (IACUC) of Central South University (Approval No. CSU‐2022‐0001‐0073).

### SPR‐LC‐MS/MS Approach

Bufalin was immobilized on a 3D photo‐cross‐linking SensorChip. Spotting was performed using a BioDots AD‐1520 Array Printer, which deposited Bufalin and control samples onto the chip surface. After spotting, the solvent was evaporated under a dark nitrogen atmosphere. The chips were then subjected to photo‐cross‐linking using a UV spectroirradiator. During the SPR assay, the mobile phase consisted of MDA‐MB‐231 cell lysates, while the stationary phase was composed of immobilized Bufalin molecules on the chip surface. The SPR biosensor system was used to monitor, in real time, the interactions between the immobilized Bufalin and potential target proteins present in the cell lysate. Following the SPR measurement, the chip was subjected to in situ trypsin digestion within the monitoring system. The resulting peptides were analyzed by HPLC‐MS to identify the proteins enriched on the chip surface.

### Cell Lines and Culture

The human triple negative breast cancer cell line HCC1806 and BT549 were cultured in RPMI‐1640 medium (Gibco, Carlsbad, CA, USA), MDA‐MB‐231 and 293T cells were cultured in Dulbecco's Modified Eagle Medium (DMEM) (Gibco, Carlsbad, CA, USA). All cell culture media were supplemented with 10% Fetal Bovine Serum and 1% penicillin‐streptomycin and were maintained at 37 °C in a humidified atmosphere of 5% CO_2_/95% air. All cell lines were tested negative for mycoplasma contamination.

### Reagents and Antibodies

Bufalin was purchased from APExBIO (Houston, TX, USA). MG‐132 was purchased from MedChemExpress (Monmouth Junction, NJ, USA). MLN4924 was purchased from TargetMol (Washington, USA). STK33 (cat. no.12857‐1‐AP), PARP1 (cat. no. 13371‐1‐AP), HSP90 (cat. no. 60318‐1‐Ig), GST (cat. no. 10000‐0‐AP), and β‐actin (cat. no. 81115‐1‐RR) antibodies were purchased from Proteintech (Chicago, IL, USA). Antibodies targeting LC3 (cat. no. 12741) and Bcl‐2 (cat. no.15071) were purchased from Cell Signaling Technology (Danvers, MA, USA). Anti‐MYC (cat. no. 100029‐MM08) was purchased from Sino Biological Inc. (Beijing, China). Anti‐Phospho‐(Ser/Thr) Phe antibody (cat. no. ab17464) was purchased from Abcam (Cambridge, England). The CCAR1 antibody (CSB‐PA816898ESR1HU) was purchased from CUSABIO (https://www.cusabio.com/). The anti‐Flag (cat. no. M185) and anti‐HA (cat. no.M180) were purchased from MBL (Medical & Biological Laboratories Co., Ltd., Nagoya, Japan), while the antibody against ubiquitin (cat. no. sc‐8017) and protein A/G agarose beads (cat. no. 10121) were obtained from Santa Cruz Biotechnology (Santa Cruz, CA, USA). Anti‐mouse and anti‐rabbit secondary antibodies were purchased from Abiowell (Shanghai, China). Streptavidin FITC (cat. no. 11‐4317‐87) was purchased from Thermo Fisher Scientific (Waltham, MA, USA), and the magnetic streptavidin beads (cat. no. 22305‐1) were purchased from Beaver (Suzhou, China). Lipofectamine 8000 was purchased from Beyotime Biotechnology (Shanghai, China). Lipofectamine RNAiMAX was purchased from Invitrogen. The CCK‐8 was purchased from Bimake (Shanghai, China). An enhanced chemiluminescence kit (cat. no. BL520A) was purchased from BioSharp (Shanghai, China).

### siRNA and Plasmid Transfection

siRNA targeting STK33 was purchased from Genepharma (Suzhou, China). The target sequence of STK33 siRNA was as follows: (1#‐GGGCUGCAGUGGAAAUCAATT UUGAUUUCCACUGCAGCCCTT, 2#‐GGAGGUGCCAAAUAAUUAUTT AUAAUUAUUUGGCACCUCCTT, 3#‐ GCCAUUAACUUGCUGCUAATT UUAGCAGCAAGUUAAUGGCTT). The STK33 plasmid was purchased from Gene (Shanghai, China). The siRNA was transfected using Lipofectamine IMAX, and the plasmid was transfected using Lipofectamine 8000 according to the manufacturer's instructions. In brief, cells in the exponential growth phase were seeded into 6‐well tissue culture plates at an appropriate density and allowed to adhere and grow for 24 h under standard culture conditions (37 °C, 5% CO_2_). After incubation, cells were transfected with a mixture containing plasmid DNA or siRNA and Lipofectamine 8000 or Lipofectamine IMAX (Invitrogen), following the manufacturer's protocol. The transfection complexes were prepared in Opti‐MEM reduced‐serum medium (Gibco) and incubated with the cells for the indicated time to ensure efficient delivery of nucleic acids.

### Western Blot

After treatment, cells were lysed on ice for 30 min using RIPA buffer supplemented with a protease inhibitor cocktail (Biotool). The lysates were then clarified by centrifugation at 12 000 × g for 15 min at 4 °C. Equal amounts of protein were separated by SDS‐PAGE and subsequently transferred onto PVDF membranes. The membranes were blocked in 5% BSA and incubated overnight at 4 °C with the appropriate primary antibodies. After washing, membranes were incubated with HRP‐conjugated secondary antibodies at room temperature for 1 h. Protein bands were visualized using enhanced chemiluminescence (ECL) detection reagents.

### Cell Viability Assays

The indicated cell lines were seeded into 96‐well plates and allowed to adhere overnight under standard culture conditions (37 °C, 5% CO_2_). The next day, cells were treated with various concentrations of Bufalin for the indicated time periods. Following treatment, cell viability was assessed using the Cell Counting Kit‐8 (CCK‐8) according to the manufacturer's instructions. Briefly, 10 µL of CCK‐8 reagent was added to each well and incubated at 37 °C for 1–2 h. Absorbance at 450 nm was measured using a microplate reader, and cell viability was calculated relative to untreated controls.

### Colony Forming Assay

HCC1806 and MDA‐MB‐231 cells, after treatment with siRNA or Bufalin, were seeded into 6‐well plates at low density and cultured under standard conditions (37 °C, 5% CO_2_) for ≈10–15 days to allow colony formation. After, the cells were gently washed with PBS, fixed with 4% paraformaldehyde for 30 min at room temperature, and stained with crystal violet solution. Excess dye was removed by rinsing with distilled water, and the plates were air‐dried.

### 5‐Ethynyl‐2’‐ deoxyuridine Assay (EdU)

The EdU assay was performed following the manufacturer's instructions. Briefly, cells were incubated with 5‐ethynyl‐2′‐deoxyuridine (EdU; RiboBio) for 2 h at 37 °C. Subsequently, cells were fixed with 4% paraformaldehyde for 30 min and permeabilized with 0.5% Triton X‐100 for 10 min at room temperature. After washing, cells were stained with 1× Apollo reaction cocktail for 30 min, followed by nuclear counterstaining with Hoechst 33342 for another 30 min at room temperature. Fluorescent images were then captured using a fluorescence microscope.

### Wound Healing Assay

To assess cell migration, cells were seeded in 6‐well plates and cultured at 37 °C. A uniform scratch was made across the cell monolayer using a sterile pipette tip. The wells were then gently washed with culture medium to remove detached cells and debris. Images of the wound area were captured immediately (0 h) and at indicated time points thereafter. The wound healing rate was calculated by measuring the reduction in the scratch width over time and analyzed accordingly.

### Transwell Assay

After transfection with STK33 siRNA, cells were suspended in serum‐free medium and seeded into the upper chamber of a transwell insert. The lower chamber was filled with medium containing 20% fetal bovine serum (FBS) as a chemoattractant. Following incubation for the indicated time, cells that migrated to the underside of the membrane were fixed with 4% paraformaldehyde and stained with crystal violet. Non‐migrated cells on the upper surface were carefully removed using cotton swabs. The migrated cells were then imaged under a microscope and quantified.

### Immunofluorescence Staining

The MDA‐MB‐231 cells seeded and treated with Biotin‐Bufalin on glass coverslip were fixed in 4% paraformaldehyde for 30 min at room temperature and blocked in 5% bovine serum albumin (BSA) for 1 h. Then, cells were incubated with anti‐STK33 antibody and streptavidin FITC at 4 °C overnight, followed by Alexa Fluor 594 dye‐conjugated anti‐rabbit IgG antibody. At the end of incubation, the cell nuclei were stained with DAPI for 2 min. The fluorescence signal was detected and captured using confocal microscopy.

### Animal Studies

Briefly, the TNBC cells MDA‐MB‐231 were injected subcutaneously into female nude mice (2×10^6^ cells in 100 µL per inoculation). Tumor sizes were measured on different days after inoculation and calculated using the equation:

(1) $$V=lw^{2}/2$$

where l: length; w: width. When the tumors were palpable, mice were alternately divided into three groups and received the indicated treatment. Tumor diameter was measured by vernier caliper every other day.

### Immunohistochemistry (IHC)

The human cancer tissue specimens and animal tissue were fixed in 4% paraformaldehyde and then embedded in paraffin. Immunohistochemistry staining using the antibodies against STK33 and CCAR1 was performed according to the manufacturer's protocols. The IHC scoring was performed according to previously published methods.^[^ ^44^ ^]^ Specifically, staining intensity was scored as follows: 0 (no staining), 1 (weak staining), 2 (moderate staining), and 3 (strong staining). The percentage of positive cells was scored as 0 (<5%), 1 (5–25%), 2 (26–50%), 3 (51–75%), and 4 (>75%). The final IHC score (range: 0–12) was calculated by multiplying the intensity score by the percentage score. Based on this composite scoring system, specimens were categorized as follows: score ≥ 8 indicated high expression, while score < 8 represented low expression.

### Protein‐Ligand Binding Conformation Modeling

The conformation of the protein‐ligand complex was modelled using Schrödinger version 2022.1. The structure of Bufalin was sourced from PubChem (https://pubchem.ncbi.nlm.nih.gov/compound/Bufalin), and ligand conformations were generated using LigPrep. The structure of the STK33 protein was predicted using the I‐TASSER server,^[^ ^45^ ^]^ since AlphaFold^[^ ^46^ ^]^ failed to predict a portion of the structure that was covered by I‐TASSER. The Glide SP protocol was employed to generate the optimal conformation of the protein‐ligand complex.

### Protein–Protein Docking Conformation Modeling

The structure of the HSP90 protein was obtained from the Protein Data Bank (PDB ID: 8GAE)^[^ ^47^ ^]^ and subjected to 15 repeats of optimization using the relax module of Rosetta (version 3.5.1).^[^ ^48^ ^]^ Since the structure of the STK33 protein was predicted using the I‐TASSER server and not from crystallography, it underwent 1000 repeats of optimization using the relax protocol. One hundred conformations were generated from each optimization, and the top‐scoring conformation from Rosetta was utilized for subsequent protein–protein complex conformation modeling. Given the similarity between the binding modes of STK33 with HSP90 and the crystal structure of HSP90‐CDK4 complex (PDB ID: 5FWL),^[^ ^49^ ^]^ the aligned structure from the crystallographic data was considered as a possible binding conformation between HSP90 and STK33.

### Classical MD Simulation and MM‐PBSA Calculations

Molecular simulations of STK33‐Bufalin, STK33‐HSP90, and STK33‐Bufalin‐HSP90 complexes were conducted using Amber22 software. The proteins and ligands were parameterized using the Amber ff19SB^[^ ^50^ ^]^ and GAFF2^[^ ^51^ ^]^ force fields, respectively. The complex systems were solvated in a TIP3P water box extending 10 Å from the protein. Chloride and sodium ions were added to neutralize the system. Subsequently, energy minimization was performed for up to 10 000 steps. The systems were then heated from 0 to 300 K over 50 ps, and pressure was increased to atmospheric pressure over another 50 ps, with a time step of 1 fs during the heating and pressurizing processes. Finally, a 100 ns production classical MD simulation was conducted with a time step of 2 fs. Trajectory analysis was carried out using CPPTRAJ. MM‐PBSA binding free energy calculations were performed using MMPBSA.py from AmberTools2023,^[^ ^52^ ^]^ extracting 100 frames uniformly from the trajectory. Additionally, residue energy decomposition was performed for the Bufalin‐STK33 complex.

### Molecular Docking

The molecular structures of STK33 domains were obtained from PDB. Amino acid gaps were automatically filled using the Home‐building program. Molecular docking was performed using Maestro 9.0, the Schrodinger program, following the standard procedures described in the manual of the software.

### Surface Plasmon Resonance (SPR) Assay

Surface plasmon resonance (SPR) assay was performed to evaluate the binding interaction between Bufalin and STK33, CLCN3, and RhoA recombinant proteins. A biomolecular microarray chip with immobilized Bufalin was assembled into the SPR instrument. Purified recombinant STK33, CLCN3, and RhoA proteins at different concentrations were prepared in PBS running buffer and injected over the chip surface. Each binding cycle included an association phase where the protein flowed over the chip, followed by a dissociation phase with running buffer. After each cycle, the chip was regenerated using glycine‐HCl buffer to remove bound proteins. The resulting sensorgrams were recorded, and binding affinities were calculated using dedicated SPR analysis software.

### Patient‐Derived Organoid

Tumor tissue samples from TNBC patients were processed for organoid culture within 2 h of collection, following established protocols.^[^ ^53^, ^54^ ^]^ Briefly, fresh specimens were enzymatically digested into single‐cell suspensions and embedded in a 3D matrix composed of 50% cold Matrigel (Corning). After 5 days of culture to allow organoid formation and maturation, the organoids were dissociated into single cells and seeded into 384‐well plates for drug treatment. Organoids were exposed to Bufalin for 4 days, after which cell viability was assessed using the CellTiter‐Glo 3D Reagent (Promega) according to the manufacturer's instructions.

### Statistical Analysis

The data was analyzed using GraphPad Prism 9.0 software. All samples represent biological replicates, and the statistical measurements are presented as mean ± SD as specified in each figure. No data were excluded from the analyses, and the experiments were randomized. The Investigators were blinded to allocation during experiments and outcome assessment. Statistical analyses were performed using Student's t‐test, one or two‐way ANOVA, depending on the experimental design. Kaplan–Meier survival analyses were used to evaluate the prognostic significance of STK33 or CCAR1 levels in TNBC patients. A *P*‐value < 0.05 was considered statistically significant.

## Conflict of Interest

The authors declare no conflict of interest.

## Author Contributions

S.J. and J.L. contributed equally to this work. Y.C. and D.C. designed the study and revised the manuscript. S.J. and J.L. performed the experiments, analyzed the experimental data, and drafted the manuscript. H.L. and C.Z. conducted molecular docking experiments. X.W., R.G., and T.J. were responsible for the construction, culture, and experimentation of the organoids. C.Z. and Z.C. performed immunohistochemistry and analyzed the results of immunohistochemistry. Z.Z. helped with data analysis.

## Supporting information



Supporting Information

## Data Availability

The data that support the findings of this study are available in the supplementary material of this article.
