# Supplementary material for: Serine/Threonine Kinase 33 as a Novel Target of Bufalin in Treatment of Triple‐Negative Breast Cancer
Source: Adv Sci (Weinh). 2025 Sep 4;12(41):e06253. doi: 10.1002/advs.202506253 (PMC12591119; doi:10.1002/advs.202506253)
Supplement: Supplementary file 1 — Supporting Information [file ADVS-12-e06253-s001.pdf]

Supporting Information

Serine/Threonine Kinase 33 as a Novel Target of Bufalin in Treatment of Triple-Negative Breast Cancer

Shilong Jiang, Junyan Liu, Hui Li, Chan Zou, Xiaoya Wan, Rong Gong, Ting Jiang, Changxin Zhong, Zonglin Chen, Zewu Zhu, Dongsheng Cao‡, Yan Cheng‡

File list

Supplementary Figures:

Supplementary Figure 1 Identification of STK33 as a binding protein of Bufalin.

Supplementary Figure 2 High STK33 expression correlates with poor therapeutic outcome and facilitates the proliferation and migration of TNBC cells.

Supplementary Figure 3 STK33 promotes TNBC cell proliferation by increasing the protein stability of CCAR1.

Supplementary Figure 4 Bufalin causes the proteasomal degradation of STK33 by destroying the STK33-HSP90 complex formation.

Supplementary Tables:

Supplementary Table 1 The kinetic parameters of Bufalin and STK33, CLCN3, RhoA binding from SPR

Supplementary Table 2 The correlation between STK33 expression and clinicopathological characteristics in tissue microarray of TNBC patients

Supplementary Table 3 The binding free energy of HSP90, STK33 and Bufalin

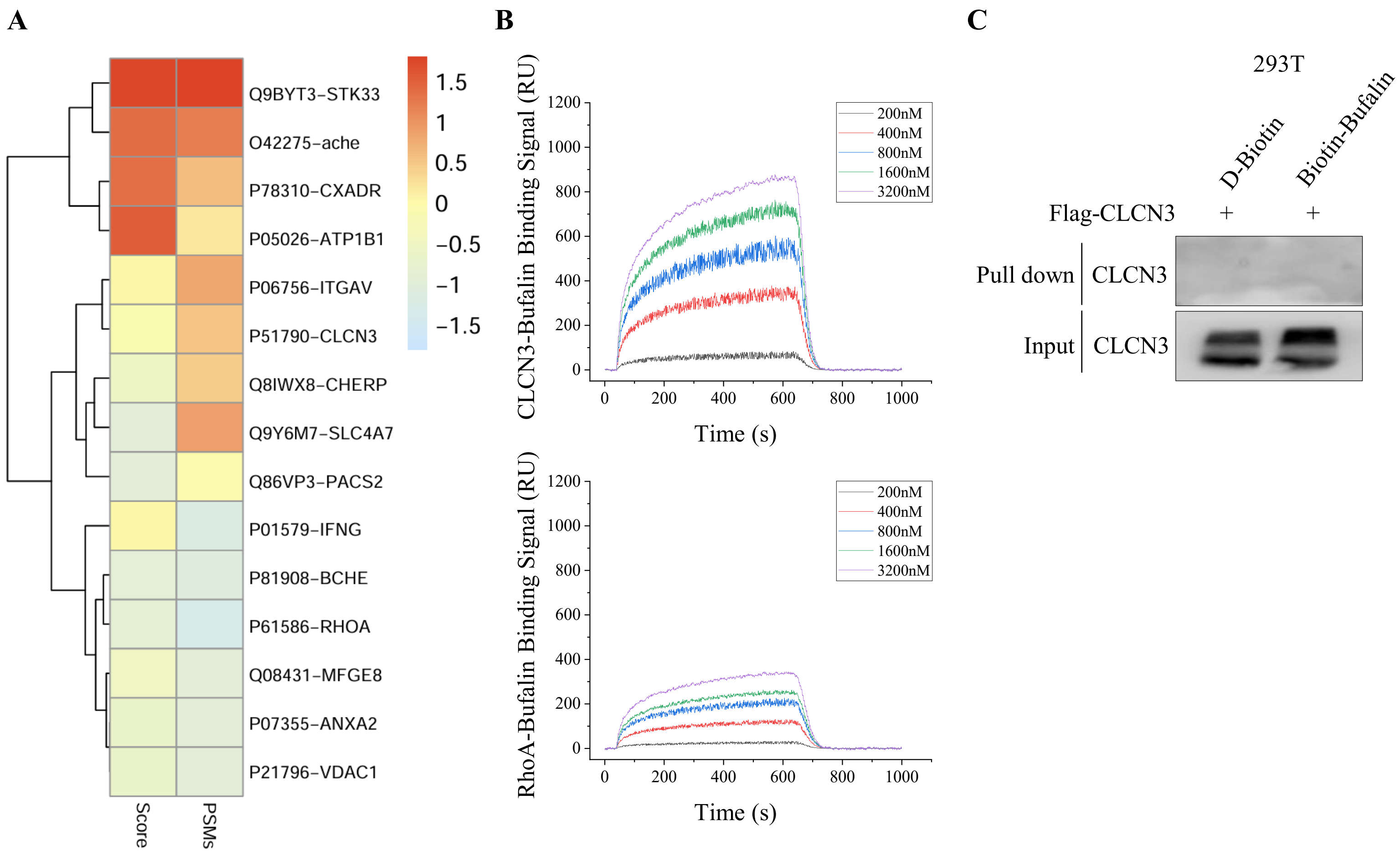

Supplementary Figure 1 Identification of STK33 as a binding protein of Bufalin. A. The top fifteen potential targets of Bufalin are presented in the relative abundance heatmap. The protein identification scores ranged from 831.01 to 1682, and the number of peptide-spectrum matches (PSMs) ranged from 12 to 84. B. SPR graph showing the interaction of Bufalin and CLCN3 or RhoA recombinant protein. C. 293T cell was transfected with Flag-CLCN3 plasmid, after transfected 48h, the cell lysates were incubated with D-Biotin or Biotin-Bufalin at 4°C overnight, followed by pull-down with streptavidin magnetic beads. The proteins bound to the magnetic beads were separated by SDS-PAGE, followed by western blot using CLCN3 antibody.

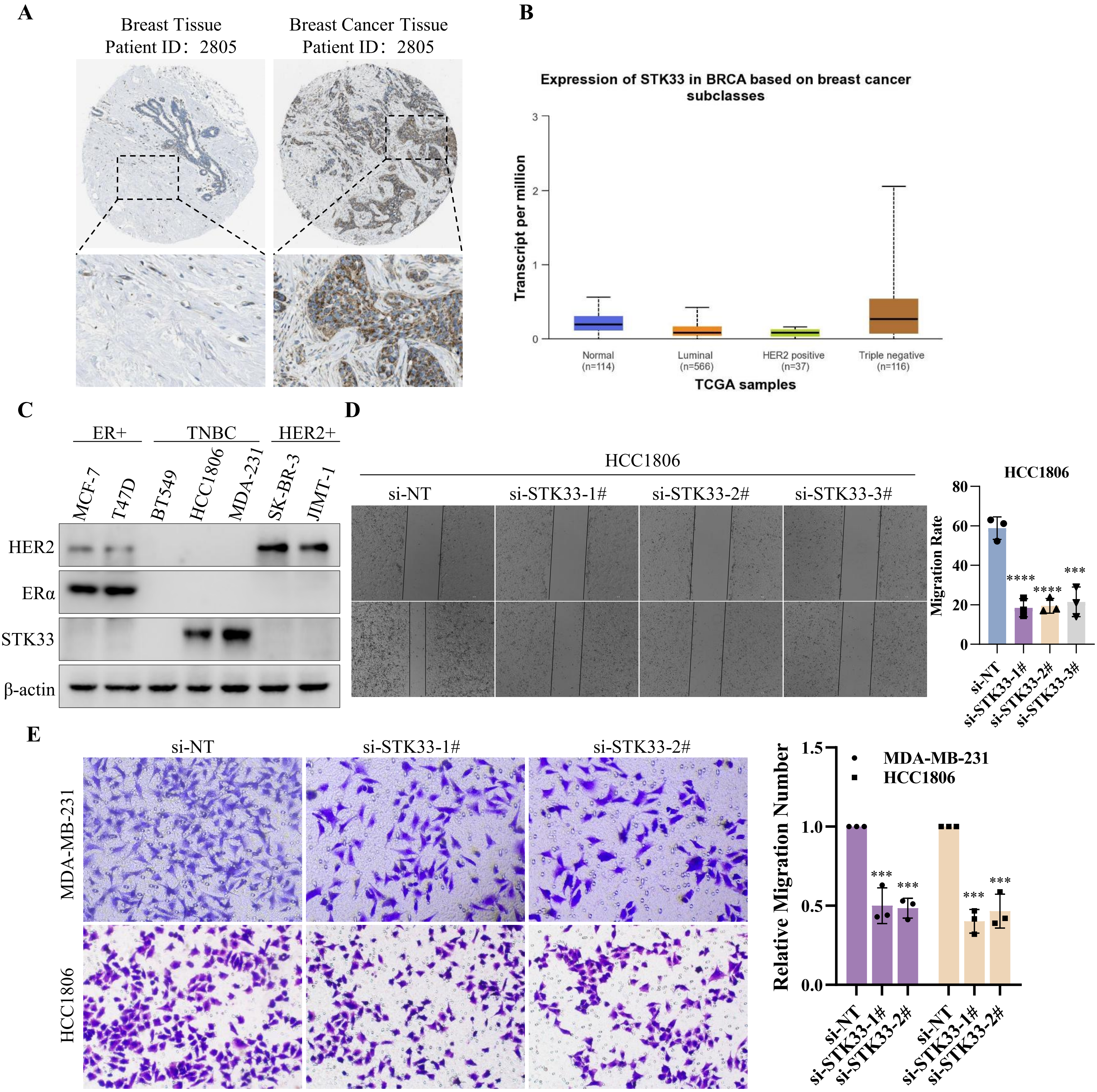

Supplementary Figure 2 High STK33 expression correlates with poor therapeutic outcome and facilitates the proliferation and migration of TNBC cells. A. Representative image of STK33 in breast cancer patient from the Human Protein Atlas (https://www.proteinatlas.org/). B. Relative expression levels of STK33 in TNBC and non-TNBC samples from the TCGA-BRCA cohort. C. The expression of STK33 in TNBC cell lines (BT549, HCC1806, and MDA-MB-231), ER positive cell lines (MCF-7 and T47D) and HER2 positive cell lines (SK-BR-3 and JIMT-1). D. Wound healing assays were performed in STK33-silenced HCC1806 cells and their corresponding negative control cells, the data are presented as mean ± SD of three independent experiments. One-way ANOVA was used to determine statistical significance, *** $P < 0.001$, **** $P < 0.0001$. E. Transwell migration assays in STK33-silenced MDA-MB-231 cells or HCC1806 cells, the data are presented as mean ± SD of three independent experiments. One-way ANOVA was used to determine statistical significance, *** $P < 0.001$.

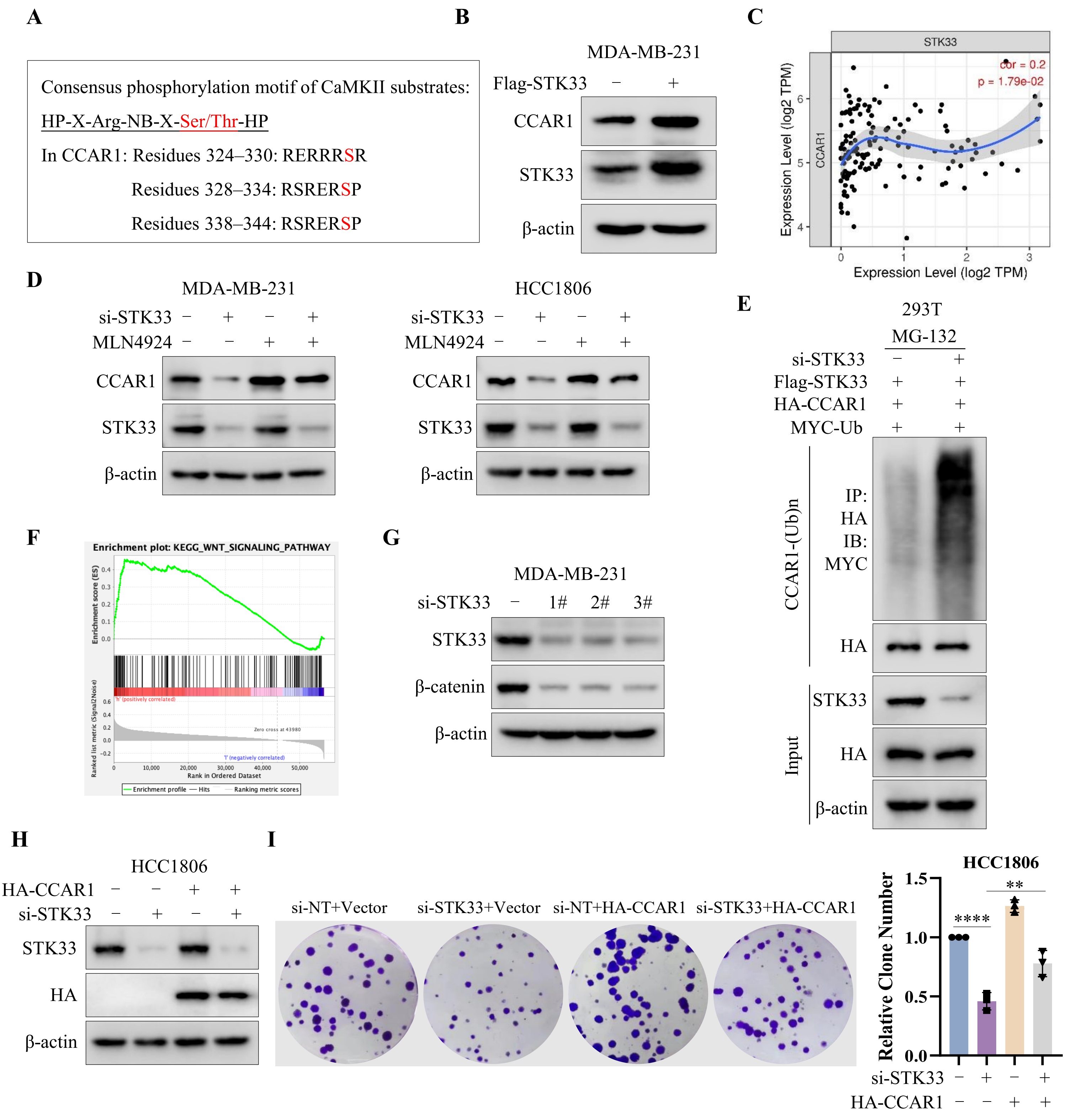
A
Consensus phosphorylation motif of CaMKII substrates:
HP-X-Arg-NB-X-Ser/Thr-HP
In CCAR1: Residues 324–330: RERRRSR
Residues 328–334: RSRERSP
Residues 338–344: RSRERSP
B
MDA-MB-231
Flag-STK33
CCAR1
STK33
β-actin
C
STK33
cor = 0.2
p = 1.79e-02
CCAR1
Expression Level (log2 TPM)
D
MDA-MB-231
HCC1806
si-STK33
MLN4924
CCAR1
STK33
β-actin
E
293T
MG-132
si-STK33
Flag-STK33
HA-CCAR1
MYC-Ub
CCAR1-(Ub)n
IP: HA
IB: MYC
HA
Input
STK33
HA
β-actin
F
Enrichment plot: KEGG_WNT_SIGNALING_PATHWAY
Enrichment score (ES)
Ranked list metric (Signal2Noise)
Rank in Ordered Dataset
Enrichment profile — Hits — Ranking metric scores
G
MDA-MB-231
si-STK33
1#
2#
3#
STK33
β-catenin
β-actin
H
HCC1806
HA-CCAR1
si-STK33
STK33
HA
β-actin
I
si-NT+Vector
si-STK33+Vector
si-NT+HA-CCAR1
si-STK33+HA-CCAR1
HCC1806
Relative Clone Number
****
**
si-STK33
HA-CCAR1

Supplementary Figure 3 STK33 promotes TNBC cell proliferation by increasing the protein stability of CCAR1. A. Schematic of the conserved substrate motif for CaMKII: HP-X-Arg-NB-X-Ser/Thr-HP (with X indicating any amino acid, NB a non-basic residue, and HP a hydrophobic residue). Candidate serine residues in CCAR1 conforming to this motif are indicated. B. MDA-MB-231 cells were transfected with Flag-STK33 plasmid, the expression of STK33 and CCAR1 was measured by western blot. C. Pearson's correlation analyses of the STK33 and CCAR1 mRNA levels in basal like breast cancer from TIMER web server (https://cistrome.shinyapps.io/timer/). D. MDA-MB-231 and HCC1806 cells were transfected with STK33 siRNA, followed by treatment with or without MLN4924. The expression of STK33 and CCAR1 was measured by western blot. E. 293T cells were transfected with HA-CCAR1, MYC-Ub, Flag-STK33 plasmids or STK33 siRNA, followed by treatment with MG132 (10 μM) for 10 hours before harvest. Then the cells lysates were subjected to immunoprecipitation with anti-HA antibody and blotted with anti-MYC antibody. F. Gene Set Enrichment Analysis (GSEA) of single gene showed that STK33 was positively correlated to Wnt/β-catenin signal pathway. G. MDA-MB-231 cells were transfected with STK33 siRNA, the expression of STK33 and β-catenin was measured by western blot. H. HCC1806 cells were transfected with STK33 siRNA, and then transfected with HA-CCAR1, the expression of STK33 and HA was measured by western blot. I. HCC1806 cells were transfected with STK33 siRNA, and then transfected with HA-CCAR1, cell proliferation was measured by the colony formation assay, the data are presented as mean ± SD of three independent experiments. One-way ANOVA was used to determine statistical significance, ** $P < 0.01$, **** $P < 0.0001$.

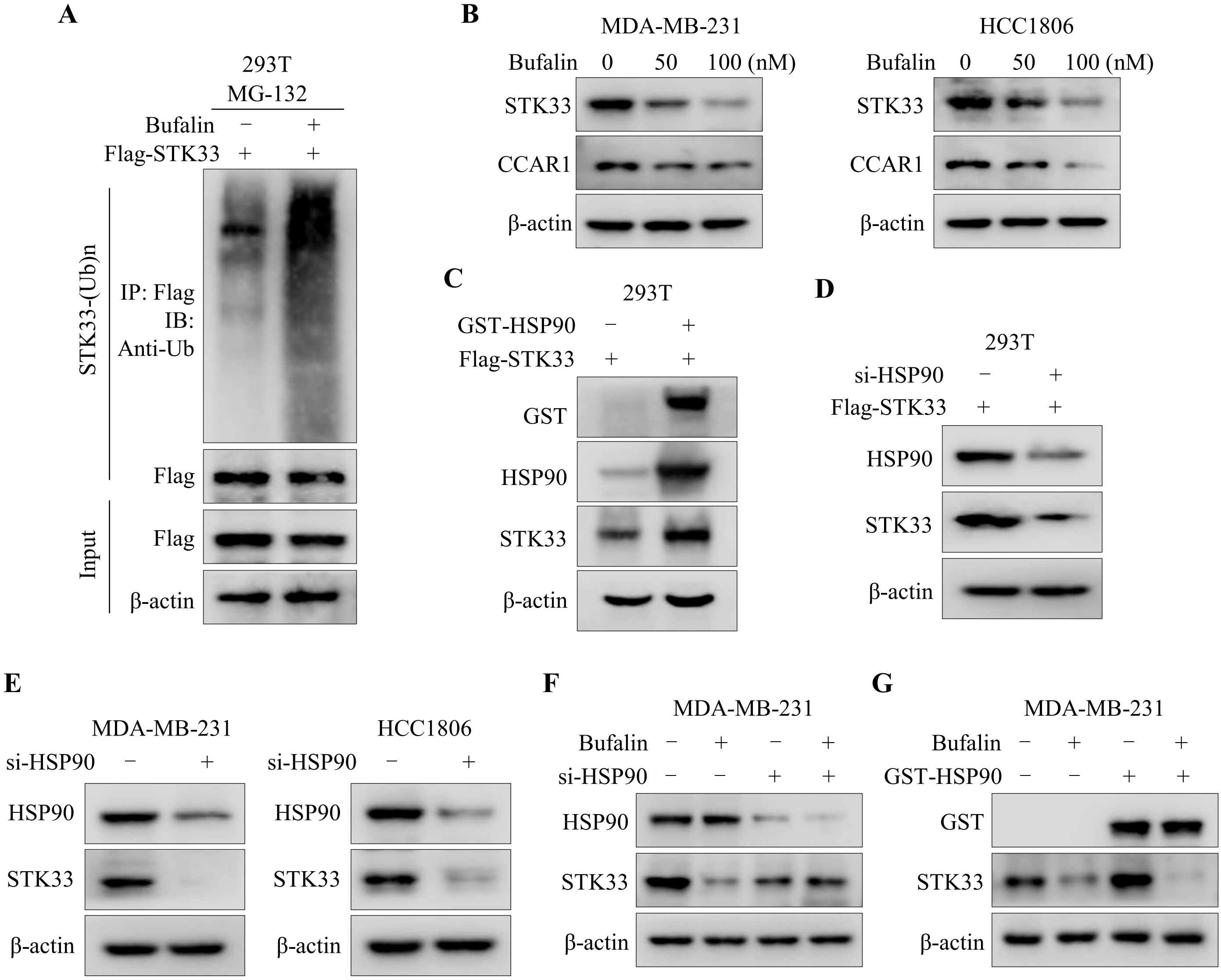

Supplementary Figure 4 Bufalin causes the proteasomal degradation of STK33 by destroying the STK33-HSP90 complex formation. A. 293T cells were transfected with Flag-STK33 plasmid, and then subjected to Bufalin for 48 h, followed by treatment with MG-132 (10μM) for 10 hours before harvest. Then the cells lysates were subjected immunoprecipitation with anti-Flag antibodies and blotted with anti-Ub antibodies. B. MDA-MB-231 and HCC1806 cells were treated with Bufalin for 48 h, CCAR1 and STK33 levels were measured by western blot. C. 293T cells were transfected with STK33 and HSP90 plasmids for 48 h. The expression of STK33 was measured by western blot. D. 293T cells were transfected with STK33 plasmid and then transfected with HSP90-targeted siRNA, the expression of STK33 was measured by western blot. E. MDA-MB-231 and HCC1806 cells were transfected with non-targeting siRNA or HSP90-targeted siRNA. The expression of STK33 was measured by western blot. F. MDA-MB-231 cells were transfected with non-targeting siRNA or HSP90-targeted siRNA followed by treatment with Bufalin for 48 h. The HSP90 and STK33 protein levels were measured by western blot. G. MDA-MB-231 cells were transfected with GST-HSP90 plasmids, followed by treatment with Bufalin for 48 h. The STK33 protein levels were measured by western blot.

Supplementary Table 1 The kinetic parameters of Bufalin and STK33, CLCN3, RhoA binding from SPR

Protein	Compound	Avg ka (1/Ms)	Avg kd (1/s)	Avg KD (M)	ABS (tr_KD)
STK33	Bufalin	3.91E+02	1.60E-04	4.10E-07	21.220
CLCN3	Bufalin	2.61E+02	3.33E-04	1.28E-06	19.580
RhoA	Bufalin	8.88E+00	5.93E-02	6.69E-03	7.225

Supplementary Table 2 The correlation between STK33 expression and clinicopathological characteristics in tissue microarray of TNBC patients

Characteristic	Total	STK33 expression		χ^2	*P*
		High 42	Low 37		
Age, years					
<50	39	22	17	0.3259	0.5681
≥50	40	20	20		
Tumor size, cm					
<3	36	19	17	0.003974	0.9497
≥3	43	23	20		
Lymph node metastasis					
Yes	44	22	22	0.3994	0.5274
No	35	20	15		
TNM stage					
I	12	3	9	4.508	0.0337
II/III/IV	67	39	28		
Ki67(%)					
<50	25	11	14	1.234	0.2667
≥50	54	31	23		

Supplementary Table 3 The binding free energy of HSP90, STK33 and Bufalin

Energy (kcal/mol)	ΔG gas		ΔG solv		Total Energy
	VDWAALS	EEL	EGB	ESURF	
**STK33-Bufalin-HSP90**	-221.4442	-454.5096	686.0359	-31.7383	-21.6760
**STK33-HSP90**	-179.3851	-128.8067	303.3138	-25.7407	-30.6261
**STK33-Bufalin**	-48.8932	-15.4870	25.7518	-6.0159	-44.6460
